# Molecular basis of surface anchored protein A deficiency in the *Staphylococcus aureus* strain Wood 46

**DOI:** 10.1371/journal.pone.0183913

**Published:** 2017-08-31

**Authors:** Manasi Balachandran, Richard J. Giannone, David A. Bemis, Stephen A. Kania

**Affiliations:** 1 Department of Biomedical and Diagnostic Sciences, The University of Tennessee, Knoxville, Tennessee, United States of America; 2 Chemical Sciences Division, Mass Spectrometry and Laser Spectrometry, Oakridge National Laboratories, Oakridge, Tennessee, United States of America; Universitatsklinikum Munster, GERMANY

## Abstract

Protein A in *Staphylococcus aureus* is encoded by the *spa* (*s*taphylococcal *p*rotein *A*) gene and binds to immunoglobulin (Ig). The *S*. *aureus* strain Wood 46 has been variously reported as protein A-deficient and/or *spa* negative and used as a control in animal models of staphylococcal infections. The results of this study indicate that Wood 46 has normal *spa* expression but transcribes very low levels of the *srtA* gene which encodes the sortase A (SrtA) enzyme. This is consistent with unique mutations in the *srtA* promoter. In this study, a low level of sortase A explains deficient anchoring of proteins with an LPXTG motif, such as protein A, fibrinogen-binding protein and fibronectin-binding proteins A and B on to the peptidoglycan cell wall. The activity of secreted protein A is an important consideration for use of Wood 46 in functional experiments and animal models.

## Introduction

Protein A produced by *S*. *aureus* is a potent virulence factor and immune-modulator [1–5]. It is a 40–60 kDa cell-wall protein encoded by the *spa* gene [6, 7]. It is synthesized by nearly all *S*. *aureus* isolates and binds to the Fc and F(ab)_2_ regions of immunoglobulins [8, 9], prevents opsonophagocytic killing [1, 10, 11] and serves as a B-cell superantigen [12–14]. Furthermore, marginal zone B cells and B-1 cells undergo, preferentially, induced cell death with supraclonal depletion and immune tolerance upon exposure to protein A [15]. Thus, protein A may interfere with vaccine efficacy that depends on opsonophagocytic antibody production by binding to B-cells and preventing their function. The *S*. *aureus* strain Wood 46 had been considered *spa* negative and protein A-deficient. For this reason, it was used as a negative control in studies involving *S*. *aureus* virulence [1, 16–22]. There is evidence that Wood 46 produces extracellular protein A during exponential growth phase and steady state [18]. This has been demonstrated indirectly by immunoglobulin binding experiments [18]. Extracellular protein A has also been isolated from Wood 46 using IgG Sepharose chromatography, although the amount of putative protein A recovered from Wood 46 is lower compared to *S*. *aureus* strain Cowan 1, a high producer of protein A [17, 18]. *S*. *aureus* produces a second immunoglobulin-binding protein (Sbi), which is also capable of binding to the Fc portion of IgG similar to protein A and protects the bacteria against innate immune responses [23].

Sortase A (SrtA) is a transpeptidase commonly produced by Gram-positive bacteria. It has specificity for proteins with an LPXTG motif and cleaves between the threonine (T) and glycine (G) residues to form an acyl enzyme intermediate that is relieved by the nucleophilic attack of the amino group in the pentaglycine cross bridge of the peptidoglycan precursor[24]. Modified proteins are subsequently incorporated into the peptidoglycan cell wall and displayed on the surface [24–30]. *S*. *aureus* isolates encode approximately 17–21 surface proteins with an LPXTG motif [4, 31, 32]. These include protein A, fibrinogen-binding proteins—clumping factors A and B (Clf A/B) and fibronectin binding proteins (FnBP A and FnBP B) that function as potent virulence factors [33]. *S*. *aureus* mutants lacking SrtA fail to covalently attach proteins with an LPXTG motif onto their cell wall, and are thus less virulent than their wild-type counterparts. They do not form abscess lesions in organs or cause bacteremia in mouse models [32, 34]. In this study, the reason for protein A deficiency on the surface of Wood 46 was investigated and the defective expression of *spa* and *srtA* genes encoding functional proteins was examined as a possible cause.

## Materials and methods

### Bacterial strains and growth conditions

*S*. *aureus* strains Wood 46 (ATCC 10832), Seattle 1945 (ATCC 25923) and Cowan 1 (ATCC 12598) were obtained from the American Type Culture Collection. The *S*. *aureus* Newman strain and its corresponding *spa* and *sbi* mutants (Newman WT, Nweman sbi::Em^r^, Newman spa::Ka^r^ and Newman sbi::Em^r^ spa::Ka^r^) were a kind gift from Drs. Tim Foster and Joan Geoghegan (Department of Microbiology, Trinity College Dublin) [23]. Bacteria were grown overnight in Trypticase Soy Broth (TSB, Becton Dickinson, Franklin Lakes, New Jersey) at 37°C in a shaker incubator and diluted 1:50 in fresh TSB to initiate log-phase cultures.

### DNA extraction and conventional PCR

Bacterial DNA was extracted from overnight cultures using a commercial kit (MoBio Ultra Clean DNA Isolation Kit, MoBio, Carlsbad, CA) according to the manufacturer’s protocol. PCR for *spa*, *srtA* and the -35 regulatory regions was performed using primers listed in Table 1. The following cycling conditions were performed: initial denaturation at 95°C for 90s, annealing at 55°C for 30s, extension at 72°C for 1min (30 cycles) and a final extension at 72°C for 5min. PCR products were sequenced at the Genomics Core Facility (University of Tennessee, Knoxville) and the sequences of *spa*, *srtA* and the -35 regulatory region were aligned and compared using Geneious (version 9.1.6) (Biomatters, Auckland, New Zealand).

**Table 1:** List of primers used in this study.

Gene	Primers	Reference
*spa* (conventional PCR)	R 5’-CGCTGCACCTAACGCTAATG-3’	This study
*srtA* (conventional PCR)	F 5’-AAGCGTTTCGTTATTTGAATGC-3’ R 5’-TCGTCATTGCTACCTCATACC-3’	This study
*spa* (qPCR)	5’-/56-FAM/TTTGTCAGC/ZEN/AGTAGTCGCGTTTGC/3IABkFQ/-3’ 5’-ATGTCGTTAAACCTGGTGATACA-3’ 5’-GGTTTGCTGGTTGCTTCTTATC-3’	This study
*srtA* (qPCR)	5’-/56-FAM/AGCAAGCTA/ZEN/AACCTCAGATTCCGAAAGA/3IABkFQ/-3’ 5’-GAACAGGCGAGTAAAGACAATAAG-3’ 5’-GGTCCTGGATATACTGGTTCTTT-3’	This study
16S (qPCR)	5’-/56-FAM/CCGGCAGTC/ZEN/AACTTA/3IABkFQ/-3’ 5’-CACCTTCCTCCGGTTTGTCA-3’ 5’-CCCTTGAACTTAGTTGCCATCATTC-3’	[35]

F 5’-ATCTGGTGGCGTAACACCTG-3’

### RNA extraction and quantitative real-time PCR

Total RNA was extracted from log phase bacterial cultures using a commercial kit (MoBio Ultra Clean RNA Isolation kit, MoBio, Carlsbad, CA) according to the manufacturer’s protocol. Quantitative Real-Time PCR (qPCR) for *spa* and *srt A* were performed using the TaqMan RNA-to-C_T_ 1-Step kit on an ABI StepOne PCR System (Applied Biosystems, Foster City, CA). The primers and probe used for the qPCR are listed in Table 1. The following cycling conditions were performed: 48°C for 30min, 95°C for 10min, 95°C for 15s, 60°C for 1min (40 cycles). qPCR data were analyzed by the ΔΔC_T_ method and normalized against 16S rRNA as the endogenous control as previously described [35].

### Protein A ELISA

Secreted protein A in bacterial culture supernatants was measured using an antigen capture ELISA. Briefly, 96-well Costar plates (Corning Inc, Corning, NY) were coated with 1μg/ml of mouse monoclonal anti-protein A antibody clone SPA-27 (Sigma-Aldrich, St. Louis, MO) in PBS pH 7.2. Bacterial supernatant was added and incubated at 37°C for 1 hour. Bound antigen was detected using a 1:500 dilution of HRP-conjugated chicken anti-protein A antibody (Gallus Immunotech, Cary, NC) followed by addition of 100μl/well of tetramethylbenzidine (TMB) substrate (Sigma-Aldrich, St. Louis, MO). Plates were washed in between each step with PBS containing 0.05% polysorbate-20 using an EL_X_405 auto plate washer (BioTek Instruments Inc., Winooski, VT). The reaction was stopped by adding 50μl/well of 0.18M sulfuric acid. The optical density was measured at 450nm using an EL_X_800 Universal Microplate Reader (BioTech Instruments Inc. Winooski, VT). Commercially available *S*.*aureus* Protein A (Thermo Scientific, Rockford, IL) was used to generate the standard curve.

### Flow cytometry to measure cell wall-associated protein A, fibrinogen-binding protein and fibronectin-binding protein

One milliliter of log phase bacterial cells were washed and incubated with either no conjugate (NC, negative control), 2μg/ml of chicken anti-protein A FITC conjugate (Gallus Immunotech, Cary, NC), 1μg/ml human fibrinogen FITC conjugate (Zedira GmbH, Darmstadt, Germany) or 1μg/ml bovine fibronectin HiLyte Fluor ^TM^ 488 conjugate (Cytoskeleton Inc., Denver, CO) for 30 min at room temperature in the dark. Excess conjugate was removed by washing and the amount of binding determined by measuring fluorescence using an Attune acoustic focusing cytometer (Applied Biosystems, Foster City, CA) at excitation/emission wavelengths of 488/519 nm.

### Statistical analysis

A one-way ANOVA and multiple comparisons using Tukey’s honest significant difference were performed to test if there was a significant difference in the expression of *spa* and *srtA* among bacterial isolates, and also to determine if there was a significant difference in the amount of surface-expressed protein A, fibrinogen-binding protein and fibronectin-binding protein. Each experiment was repeated at least three times and a *p-value* of <0.05 was considered significant. All analyses were conducted using the IBM SPSS Statistics for Windows, Version 22.0 (IBM Corp, Armonk, NY).

## Results

### Wood 46 expresses lower amounts of cell wall-associated protein A

**Fig 1:**
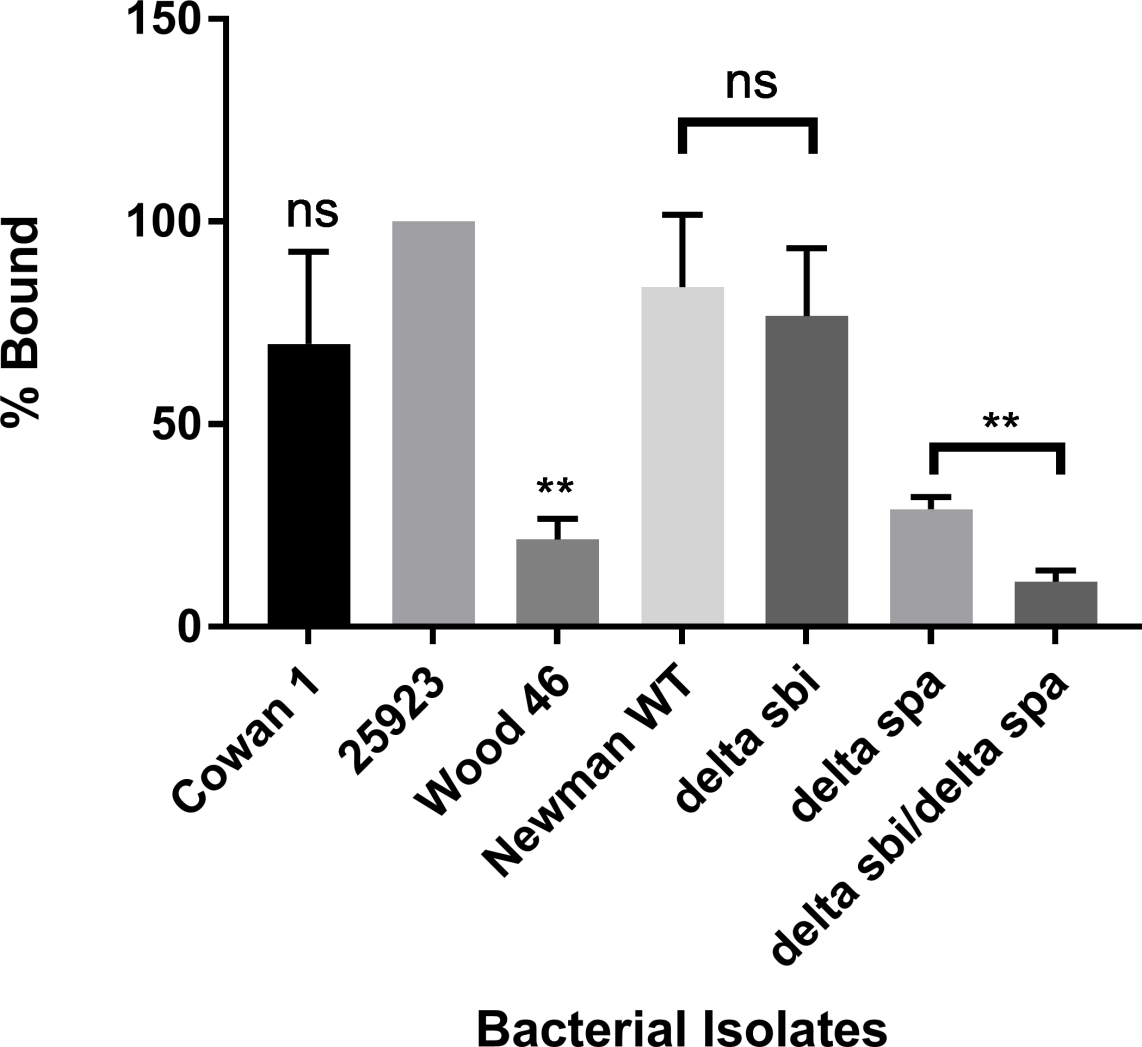
Cell wall-associated protein A. Protein A expressed on the surface of *S*. *aureus* strains Cowan1, Seattle 1945 (25923), Wood 46, Newman WT and its corresponding deletion mutants, *Δ*sbi, *Δ*spa and *Δ*sbi-*Δ*spa, as measured by flow cytometry with chicken anti-protein A antibody. The values represent the average from three independent experiments. (*P<0.05, **P<0.02, ***P<0.01, ****P<0.001).

The *S*. *aureus* isolate Wood 46 had been reported as *spa*-negative and protein A-deficient although there have been other studies that demonstrated that it binds small quantities IgG suggesting that it may have a low density of protein A on its surface [16–22]. Flow cytometry using chicken anti-protein A antibody was performed to determine the amount of cell wall-associated protein A. The amount of binding was normalized against Seattle 1945 which showed maximum binding in this assay (assigned 100% binding). Wood 46 bound lower amounts of the antibody (about 19%) relative to Seattle 1945. The *Δ*sbi, *Δ*spa and *Δ*sbi-*Δspa* mutants bound 76%, 27% and 9% of the antibody respectively. In contrast, Cowan 1 and the Newman WT isolate, both reported to be high producers of Protein A bound 69% and 83% respectively (Fig 1), indicating that Wood 46 may have lower amounts of cell wall-associated protein A compared to the control strains used in this study.

### Secreted protein A in culture supernatant of Wood 46

**Fig 2:**
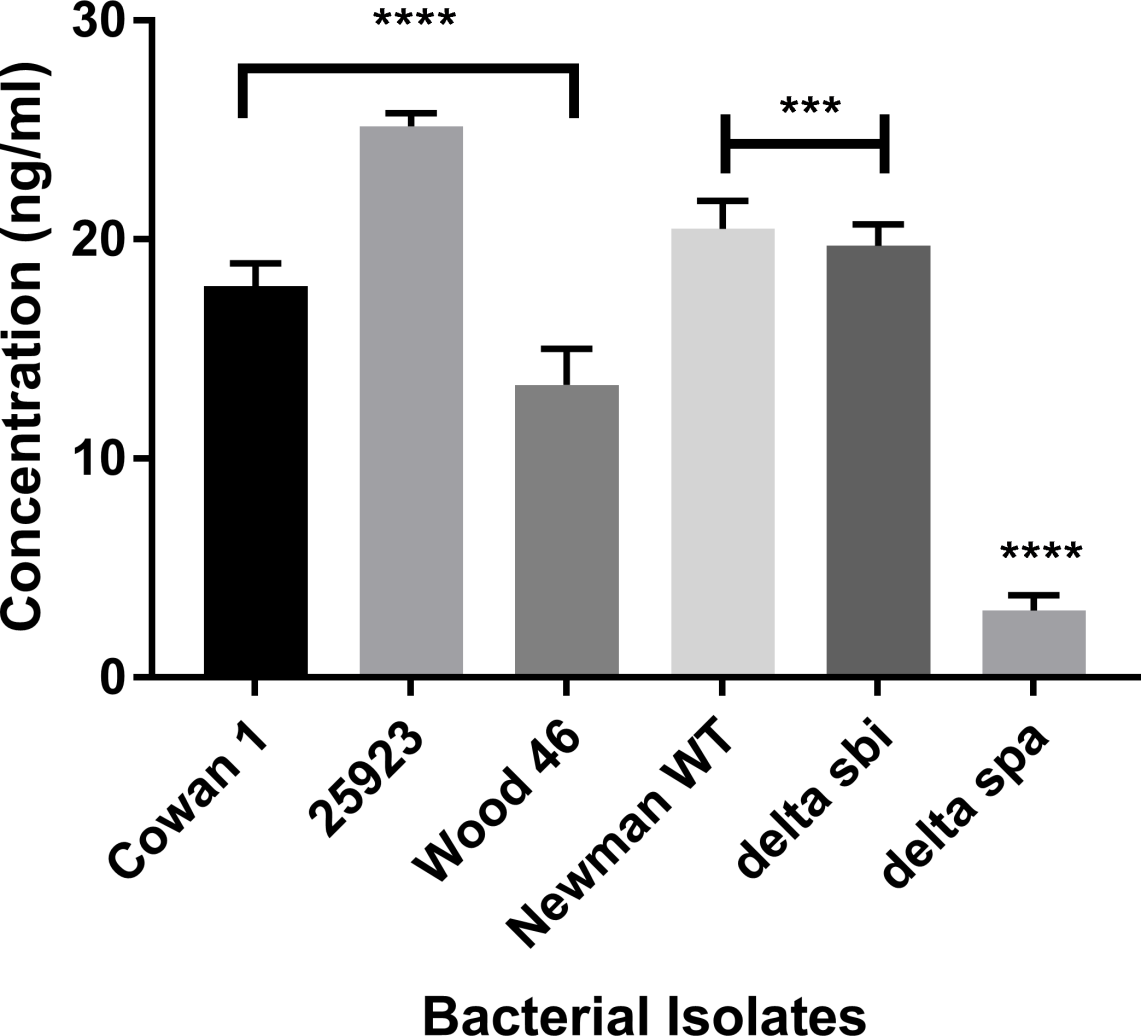
Production of extracellular protein A in *S*. *aureus*. The amount of secreted protein A was measured using an antigen-capture ELISA. Commercially available protein A from *S*. *aureus* was used to generate the standard curve. The values represent the average from three independent experiments. (*P<0.05, **P<0.02, ***P<0.01, ****P<0.001).

*S*. *aureus* releases protein A during the exponential growth phase [36, 37]. To confirm this in Wood 46, a protein A-capture ELISA was used to measure the amount of protein A secreted into culture supernatant. Culture supernatants from bacteria grown to log phase were tested. Wood 46 secreted 12.42ng/ml of protein A into the culture supernatant compared to Seattle 1945 which secreted the highest amount of Protein A (22.56ng/ml) during log phase. The Cowan 1, Newman WT and the *Δ*sbi produced 16.68ng/ml, 18.78ng/ml and 17.97ng/ml of protein A (Fig 2). These data indicate that Wood 46 produces and secretes protein A, although the amount is lower compared to Seattle 1945 or wild-type Newman isolates. The lower limits of detection and quantification of the assay were 0.125ng/ml and 0.3ng/ml respectively.

### Gene sequence analysis of *spa*, *srtA* and regulatory region of *srtA*

It was hypothesized that if the *spa* gene in Wood 46 contains a mutation, it may lead to a truncated or non-functional protein. In order to test this hypothesis, the *spa* gene in Wood 46 and control *S*. *aureus* isolates was amplified using PCR and the products were sequenced and analyzed. Results from the PCR and DNA sequence analysis of the *spa* gene showed that Wood 46 not only possesses the full-length *spa* gene, but also the sequence of the *spa* gene is in agreement with those of Cowan 1, Seattle 1945 and Newman WT. Therefore, mutation in the *spa* gene was ruled out as the possible cause for lower expression of Protein A in Wood 46.

**Fig 3:**
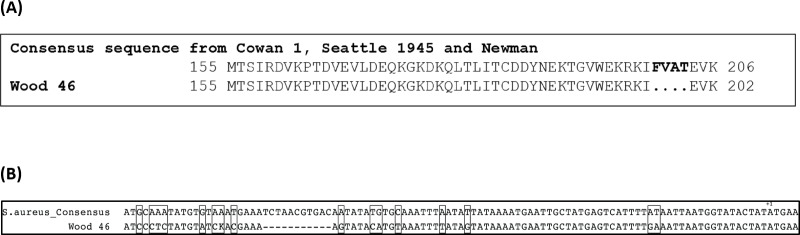
**(A) Amino acid composition of sortase A.** The amino acid sequence of sortase A in Cowan 1, Seattle 1945, Newman and Wood 46 were aligned. Amino acids from position 155 to the end of the protein are shown. Wood 46 contains a 12bp deletion at the 3’ end of the *srtA* gene that leads to a corresponding loss of four amino acids at the C-terminus of the protein. **(B). Promotor and upstream regulatory region of *srtA* in Wood 46**. Consensus promoter and upstream regulatory sequence from Cowan 1, Seattle 1945 and Newman was aligned with that of Wood 46. The boxes highlight regions with mutations. The transcription start site ATG is indicated as +1.

Sequence analysis of the *srtA* gene in Wood 46, however, showed that it has a 12bp deletion at the 3’ end of the gene that results in a four amino acid difference in the protein compared to *S*. *aureus* Cowan 1, Seattle 1945 and wild-type Newman (Fig 3A). Also, analysis of the upstream promoter and regulatory region showed that Wood 46 contains mutations in both the -10 and -35 regions (Fig 3B). These mutations likely adversely affect RNA polymerase binding and transcription as mutations in the upstream promoter and regulatory regions have been associated with reduced gene expression [38–40].

### mRNA levels of *spa* and *srtA* in Wood 46

**Fig 4:**
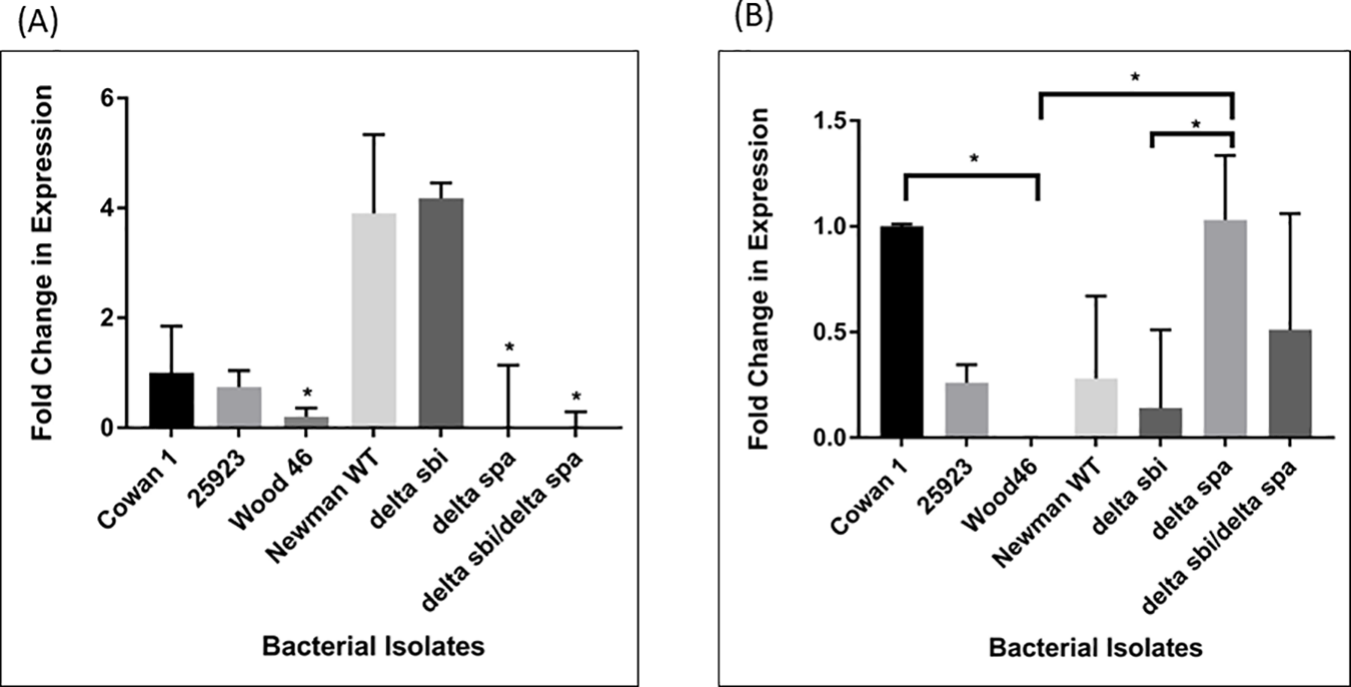
Expression levels of *spa* and *srtA*. Quantitative real-time PCR was used to measure the amount of (A) *spa* mRNA and (B) *srtA* mRNA in log phase bacterial cells. Relative gene expression was calculated using the ΔΔC_T_ method and expressed as fold change. 16S rRNA was used as the endogenous control. The amount of *srtA* mRNA in Wood 46 was below the detection limit of the assay. The values represent the average from three independent experiments. (*P<0.05, **P<0.02, ***P<0.01, ****P<0.001). ND- not detected.

If the transcription of *spa* is adversely affected, it could lead to lower expression of protein A on the bacterial surface. Reverse transcriptase qPCR was used to analyze the expression of *spa* in *S*. *aureus* isolates. The results showed that Wood 46 transcribes *spa* mRNA although the levels were lower compared to Cowan 1 which was assigned as the calibrator strain for this experiment (Fig 4A). The expression levels of *spa* in Seattle 1945, Newman WT and *Δ*sbi were 0.74 fold, 3.9 fold and 4.18 fold respectively. As expected, the *Δ*spa and *Δ*sbi-*Δspa* mutants did not produce detectable *spa* mRNA. This suggests that inefficient *spa* transcription was not the reason for low surface expression of Protein A in Wood 46.

Conversely, the expression level of *srtA* mRNA in Wood 46 was below the limit of detection of the assay. Cowan 1 was assigned as the calibrator strain for this experiment. The expression levels of Seattle 1945 and Newman WT were 0.26 fold and 0.28 fold relative to Cowan 1, while that of the deletion mutants *Δ*sbi, *Δ*spa and *Δ*sbi,*Δ*spa were 0.14 fold, 1.03 fold and 0.51 fold respectively (Fig 4B). Taken together, the results suggest the lack of sortase A could be responsible for the deficient anchoring of protein A in Wood 46. The *spa* and *srtA* expression levels were normalized against 16S rRNA as the endogenous control and the results were expressed as fold change in mRNA levels using the ΔΔC_T_ method. To ensure equal amplification efficiencies of the target and control genes, four 10-fold serial dilutions of the target RNA were tested. The efficiencies of both the target gene and the endogenous control were comparable.

### Wood 46 expresses lower amounts of the LPXTG motif-containing fibrinogen-binding protein and fibronectin-binding protein

**Fig 5:**
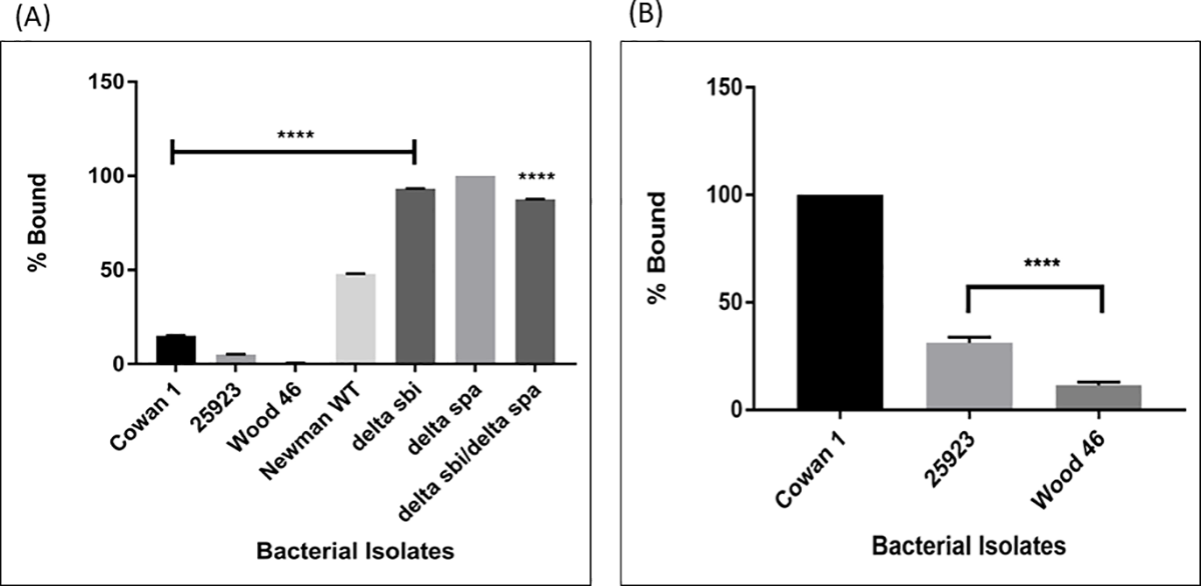
Fibrinogen-binding and fibronectin-binding assay. The amount of sortase A-anchored, LPXTG-motif containing fibrinogen-binding protein (A) and fibronectin-binding protein (B) was measured by flow cytometry using FITC-conjugated fibrinogen and HiLyte Fluor ^TM^ 488-conjugated fibronectin respectively. The percentage binding was determined relative to highest value obtained for that experiment. The values represent the average from three independent experiments. (*P<0.05, **P<0.02, ***P<0.01, ****P<0.001). *(Since the Newman isolate has truncations in both FnBP-A and FnBP-B*, *it was not tested in the fibronectin binding assay)*.

Sortase A is responsible for anchoring proteins with an LPXTG motif on to the peptidoglycan cell well [24, 27, 28, 30, 41]. If lack of this enzyme is indeed the reason for low surface density of protein A in Wood 46, there should be a global decrease in the expression of all surface proteins that bear the LPXTG motif. In order to test this hypothesis, two well-studied proteins with LPXTG motifs were chosen. Fibrinogen-binding protein (Clf A and B) and fibronectin-binding protein (FnBP A and B) are anchored on the bacterial surface and known immune evasion factors in *S*. *aureus*. A binding assay was performed with FITC-labeled human fibrinogen to detect surface-expressed fibrinogen-binding protein, and HiLyte Fluor ^TM^ 488 conjugated bovine fibronectin to detect surface-expressed fibronectin-binding protein. Flow cytometry results showed that Wood 46 bound only 0.49% of labeled fibrinogen on its surface compared to the *Δ*spa isolate which bound the highest amount of fibrinogen in this study (Fig 5A). Also, Wood 46 bound only 11.4% of the labeled fibronectin relative to Cowan 1 (considered as 100%) and Seattle 1945 (31.2%) (Fig 5B). The Newman isolate and its corresponding deletion mutants were not tested in the fibronectin-binding assay because they have truncations in both FnBP A and FnBP B [42]. These data suggest that lack of sortase A in Wood 46 likely results in lower amounts of not just protein A, but other proteins that contain the LPXTG motif.

## Discussion

*S*. *aureus* produces a number of virulence factors including secreted toxins, enzymes and cell wall-associated proteins that bind to and interact with host extracellular matrix [43]. In an *in vitro* environment, the production of virulence factors is growth-phase dependent [44]. The secreted proteins are produced during post-exponential (stationary) phase wheras the cell wall-associated proteins are predominantly produced during the logarithmic growth phase [45, 46]. Many of the known virulence factors such as protein A, fibrinogen-binding proteins like clumping Factors A and B, fibronectin-binding proteins A and B are synthesized and anchored onto the peptidoglycan cell wall by the transpeptidase enzyme, sortase A [27, 30, 41, 47].

The *S*. *aureus* isolate Wood 46 has played an important role in studies designed to examine the biological function of protein A and its role in pathogenesis and virulence. There have been contradictory reports on the production, display and secretion of protein A [18, 48]. The results have either been ambiguous or lacked definitive experimental data. Wood 46 has been described as a *spa*-negative and protein A-deficient strain although there is evidence that Wood 46 expresses low amounts of protein A on its surface as evidenced by its ability to bind low amounts of IgG [18, 49]. It is also reported that Wood 46 secretes small amounts protein A into the culture supernatant during exponential growth phase [17, 18]. Recently, several mutant strains have replaced Wood 46 as the preferred choice for negative control in both *in vitro* and *in vivo* studies. However, the mechanism for low expression of protein A on the surface of Wood 46 had not been previously investigated.

Cell wall-associated protein A binds to IgG via its Fc region, thereby preventing it from opsonization and phagocytosis [50]. In this study, it was observed that Wood 46 had a lower density of protein A on its surface, and secreted less protein A compared to the Cowan 1, Seattle 1945 or Newman isolates. It was observed that even the *Δ*spa isolate bound some anti-protein A antibody. This could be due to the ability of the antibody to react with other immunoglobulin-binding proteins, such as Sbi, on the surface of the bacterium. Secreted protein A functions as a superantigen (SAg) [13]. It binds to VH_3_-clan B-cell receptors and limits their abilities to produce antibody. Also, marginal zone B-cells and B1-cells undergo apoptosis upon exposure to protein A [13–15, 29, 51, 52]. There have been reports of secreted protein A in Wood 46 activating human B-cells in a mechanism that is different from that seen in Cowan 1 [53]. Therefore, it is important to consider the toxic effects of secreted protein A, if Wood 46 is used as a control in animal models to test vaccine efficacy.

Conventional PCR and sequencing showed that Wood 46 possesses the *spa* gene with a complete open reading frame. The gene sequence is in agreement with that of Seattle 1945, Cowan 1 and Newman. qPCR for *spa* expression showed that Wood 46 does transcribe *spa* mRNA but at lower amounts compared to that of Cowan 1, Seattle 1945 and Newman isolates. Because this did not explain the low surface levels of protein A, the transpeptidase enzyme sortase A responsible for anchoring surface proteins on the peptidoglycan cell wall, was examined. Conventional PCR with *srtA*-specific primers showed that Wood 46 contains the *srtA* gene with a complete open reading frame. However, it harbors a 12bp deletion at the 3’-end of the gene resulting in a corresponding loss of four amino acids at the N-terminus of the protein sequence. It is not known if the loss of the amino acids affects the function of the protein in any way. Further analysis of the upstream region of the *srtA* gene shows mutations in the -10 and -35 regions of the promoter and regulatory that would be expected to adversely affect binding of RNA polymerase and transcription. The functional effects of these mutations are yet to be evaluated and may be pursued at a later stage. Promoter site mutations have been associated with impaired transcription and in turn affect protein expression [38–40, 54–57]. These mutations could affect the expression of *srtA* which in turn may be responsible for the low surface density of protein A. If this is true, there should be a decrease in the expression of other surface proteins that harbor the LPXTG motif. This was confirmed by examining the surface expression of two known LPXTG proteins, fibrinogen-binding protein (Clf A and Clf B) and fibronectin-binding protein (FnBP A and FnBP B). Both these proteins in *S*. *aureus* are virulence factors similar to protein A [24, 27, 29–31, 58]. Wood 46 expressed very low amounts of both fibrinogen-binding protein and fibronectin-binding protein on its surface suggesting that it may express lower amounts of all surface proteins that have an LPXTG motif. Previous studies have shown that a portion of SpA is released into the supernatant with an intact sorting signal, and release of SpA was reduced when the native sorting signal of SpA was replaced with the corresponding region of another sortase-anchored protein, namely, SdrE [59]. Since replacing the native sorting signal and determining its effects on secreted protein A were beyond the scope of this study, we instead addressed this concern by examining the sequence of secreted protein A in Wood 46 and one of the control strains, Cowan 1 by mass spectroscopy. In both Cowan 1 and Wood 46, we found peptide hits that matched with the LPETG motif suggesting that it is indeed the low level of sortase A in Wood 46 that is responsible for the low surface level of LPXTG proteins in this strain (S1 Fig). The protein abundance from mass spectrometry in each strain showed that Cowan 1 had a higher amount of protein A in the supernatant compared to Wood 46 (S1 Table) which could be correlated to the ELISA data. LytM triggers polypeptide release from the bacterial envelope. These released proteins have a modified anchor structure [36]. If LytM is inhibited, then there should be lower amounts of released proteins with the modified anchor structure. We confirmed this by mass spectrometry. We obtained peptide hits to protein A with an LPETG motif both in Cowan 1 and Wood 46 suggesting that it was indeed low amounts of sortase A in Wood 46 that is responsible for defective anchoring of protein A on the bacterial cell wall (S2 and S3 Figs). N-acetylglucosamine is a known inhibitor of LytM [60]. If LytM was inhibited, there would be decrease in the amount of secreted protein A in bacterial culture supernatants. In order to test this, bacterial strains Cowan 1 and Wood 46 were grown to mid log-phase in the presence and absence of 10mM *N*-acetylglucosamine. The amount of secreted protein A was measured by capture ELISA. It was found that although there was a slight reduction in the amount of protein A produced in each strain, the difference was not significant. If murein wall hydrolases were responsible for defective anchoring/secretion of protein A, there would have been a significant difference in the amount of cell wall-associated and secreted protein A in both Cowan 1 and Wood 46. Since this was not the case, it was concluded that the low level of sortase A in Wood 46 is responsible for defective anchoring of protein A on the surface of Wood 46. Taken together, the results strongly suggest that low amounts of sortase A are responsible for impaired surface expression of proteins with an LPXTG motif.

The complete coding sequence of the Wood 46 *srtA* gene along with the upstream promoter and regulatory region has been deposited in GenBank (accession number: KY514391). The whole genome sequence of Wood 46 has been determined and deposited in GenBank (accession number: MTFQ00000000, version MTFQ00000000.1). This could provide a wealth of information about other immunoglobulin- binding proteins with or without an LPXTG motif. It will also help identify novel genes whose products may be involved in immune evasion. Sequence information about promoters and regulatory elements of virulence genes could help explain why some strains of *S*. *aureus* are innately less virulent compared to other highly virulent strains.

In conclusion, the results from this study suggest that defective expression of *srtA* in Wood 46 accounts for the failure of protein A anchoring to its surface with the majority of the protein A being secreted. This may be accompanied by deficient anchoring of all LPXTG-bearing surface proteins. The Wood 46 promoter at both the -10 and -35 regions contained mutations compared to the control strains used in this study. Promotors that vary from consensus sequences generally are associated with decreased gene expression [54, 55, 57]. Understanding the nature of surface protein expression and its role in virulence is crucial towards successful vaccine development against *S*. *aureus*.

## Supporting information

S1 Fig Median-centered protein abundance distributions between strains as assessed by LC-MS/MS-based proteomics. Protein abundance values follow a log-normal distribution and thus were transformed (log2) prior to normalization and median-centering. Protein A abundance was denoted (orange dotted line) in each sample. Protein A exhibited a consistent abundance across technical replicates but varied widely across strains. Though it was detected in Wood 46 supernatant, the protein was only 0.46% of the abundance observed in Cowan where it was one of the most abundant protein observed.
(TIF)

S2 Fig Sequence coverage of IgG-binding protein A. Identified peptides were mapped onto the FASTA sequence of protein A to show its overall sequence coverage. Red M’s represent oxidized methionine residues. The number of underlines indicate peptide overlap and thus overall regional sequencing depth. The LPXTG region is highlighted in yellow and was identified by one peptide per Cowan replicate LC-MS/MS run.
(TIF)

S3 Fig MS/MS spectrum identifying the peptide containing the LPXTG motif. The large, multiply charged peptide: VVDKKQPANHADANKAQALPETGEENPFIGTTVF (+4) was identified only in Cowan supernatant. The intact peptide mass measured was -0.0030 Da from the peptide’s theoretical mass (-0.83 ppm) with matching fragment ions to confirm its identity. Matching b and y-ions (major series) are shown in (A) while matching a- and x-, b- and y-, and c- and z-ions (complete series) are shown in (B).
(TIF)

S1 Table Protein abundance from mass spectrometry in Cowan 1 and Wood 46 culture supernatant. Protein A was readily detected in the culture supernatant of both Cowan 1 and Wood 46 but at higher abundance in Cowan 1.
(PDF)
