# Supplementary figures and images for: Molecular basis of surface anchored protein A deficiency in the Staphylococcus aureus strain Wood 46
Source: PLoS One. 2017 Aug 31;12(8):e0183913. doi: 10.1371/journal.pone.0183913 (PMC5578664; doi:10.1371/journal.pone.0183913)

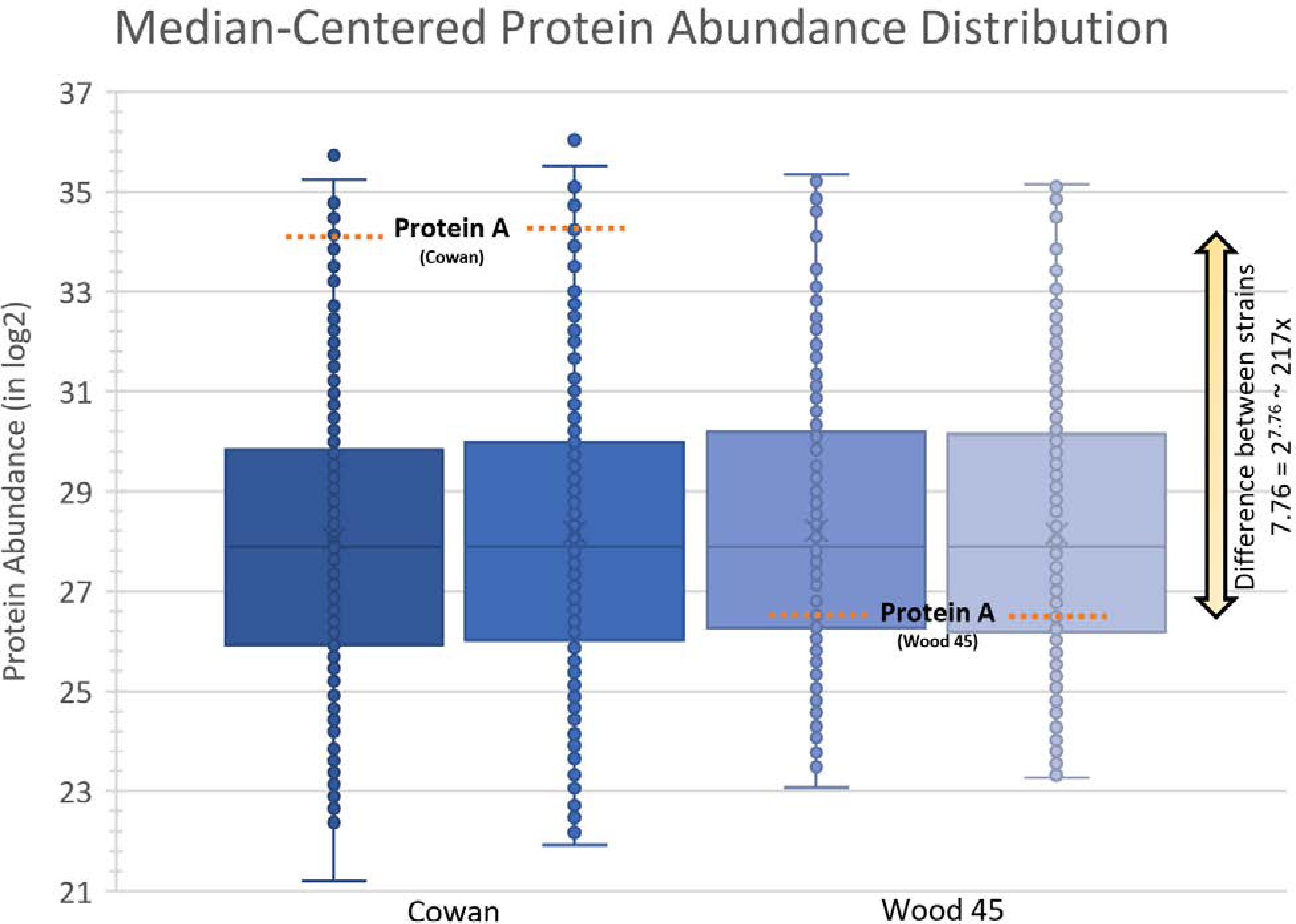

Supplement: S1 Fig — Protein abundance values follow a log-normal distribution and thus were transformed (log2) prior to normalization and median-centering. Protein A abundance was denoted (orange dotted line) in each sample. Protein A exhibited a consistent abundance across technical replicates but varied widely across strains. Though it was detected in Wood 46 supernatant, the protein was only 0.46% of the abundance observed in Cowan where it was one of the most abundant protein observed. (TIF) [file pone.0183913.s001.tif]

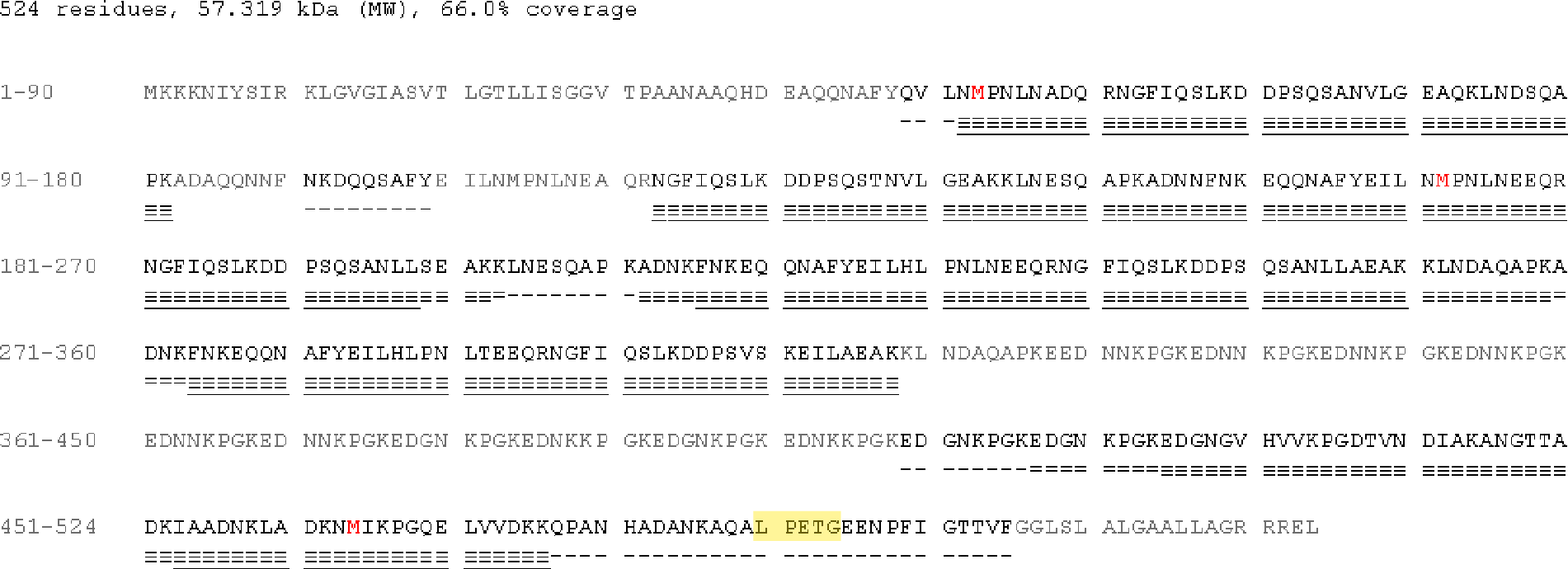

Supplement: S2 Fig — Identified peptides were mapped onto the FASTA sequence of protein A to show its overall sequence coverage. Red M’s represent oxidized methionine residues. The number of underlines indicate peptide overlap and thus overall regional sequencing depth. The LPXTG region is highlighted in yellow and was identified by one peptide per Cowan replicate LC-MS/MS run. (TIF) [file pone.0183913.s002.tif]

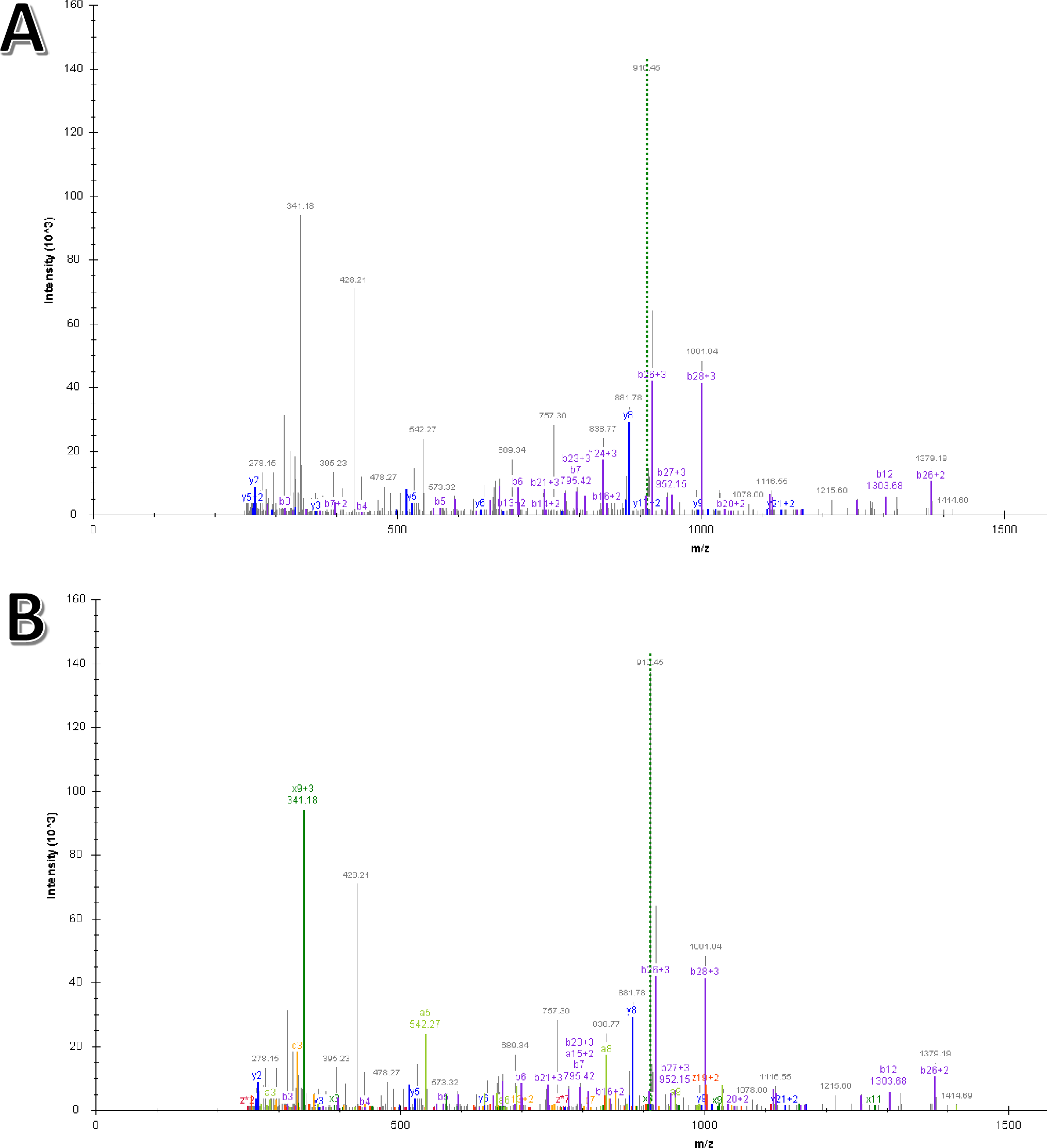

Supplement: S3 Fig — The large, multiply charged peptide: VVDKKQPANHADANKAQALPETGEENPFIGTTVF (+4) was identified only in Cowan supernatant. The intact peptide mass measured was -0.0030 Da from the peptide’s theoretical mass (-0.83 ppm) with matching fragment ions to confirm its identity. Matching b and y-ions (major series) are shown in (A) while matching a- and x-, b- and y-, and c- and z-ions (complete series) are shown in (B). (TIF) [file pone.0183913.s003.tif]
