# Supplementary material for: Molecular basis of surface anchored protein A deficiency in the Staphylococcus aureus strain Wood 46
Source: PLoS One. 2017 Aug 31;12(8):e0183913. doi: 10.1371/journal.pone.0183913 (PMC5578664; doi:10.1371/journal.pone.0183913)
Supplement: S1 Table — Protein A was readily detected in the culture supernatant of both Cowan 1 and Wood 46 but at higher abundance in Cowan 1. (PDF) [file pone.0183913.s004.pdf]

| Accession | SeqCov (%) | Peptides | Filtered Spectra | Description | Normalized Protein Abundance | | | |
|---|---|---|---|---|---|---|---|---|
| | | | | | Cowan_1 | Cowan_2 | Wood_1 | Wood_2 |
| contam_sp\|P04264\|K2C1_HUMAN | 67.7 | 102 | 1014 | Keratin, type II cytoskeletal 1 (Cytokeratin 1) (K1) (CK 1) (67 kDa cytokeratin) (Hair alpha prote | 35.7282 | 36.041 | 34.861 | 34.8523 |
| sp\|Q2G028\|ENO_STAA8 | 91.2 | 74 | 817 | Enolase OS=Staphylococcus aureus (strain NCTC 8325) GN=eno PE=1 SV=1 | 35.2467 | 35.5307 | 33.2414 | 33.2152 |
| sp\|Q2FZL2\|SSPA_STAA8 | 83.3 | 60 | 766 | Glutamyl endopeptidase OS=Staphylococcus aureus (strain NCTC 8325) GN=sspA PE=1 SV=1 | 26.0594 | 26.6582 | 35.2596 | 35.1355 |
| contam_gi\|265412\|gb\|AAB25337.1\| | 74.3 | 49 | 734 | V8 protease [Staphylococcus aureus, Peptide, 276 aa] MW: 29990.36 pI: 4.50 | 25.9206 | 26.5218 | 35.2083 | 35.0948 |
| tr\|Q2FYT8\|Q2FYT8_STAA8 | 92.0 | 116 | 718 | Transketolase OS=Staphylococcus aureus (strain NCTC 8325) GN=SAOUHSC_01337 PE=3 SV=1 | 34.5311 | 34.7304 | 32.6797 | 32.5297 |
| sp\|Q2FZL3\|SSPB_STAA8 | 71.0 | 85 | 562 | Staphopain B OS=Staphylococcus aureus (strain NCTC 8325) GN=sspB PE=1 SV=1 | | | 34.6011 | 34.4936 |
| contam_sp\|P13645\|K1CJ_HUMAN | 60.2 | 75 | 524 | Keratin, type I cytoskeletal 10 (Cytokeratin 10) (K10) (CK 10) - Homo sapiens (Human). MW: 5! | 34.0415 | 34.2426 | 33.2529 | 32.9179 |
| ProA_Target,sp\|P02976\|SPA_STAA8 | 66.5 | 92 | 522 | Immunoglobulin G-binding protein A OS=Staphylococcus aureus (strain NCTC 8325) GN=spa P | 34.1394 | 34.3992 | 26.6195 | 26.3964 |
| tr\|Q2G032\|Q2G032_STAA8 | 90.8 | 63 | 512 | Glyceraldehyde-3-phosphate dehydrogenase OS=Staphylococcus aureus (strain NCTC 8325) G | 34.7761 | 35.0954 | 32.3305 | 32.2748 |
| contam_gi\|228896\|prf\|\|1814271A | 59.9 | 45 | 504 | Glu-C endoprotease MW: 38664.17 pI: 4.99 | 25.874 | 26.6406 | 34.9371 | 34.9502 |
| sp\|Q2G1X0\|HLA_STAA8 | 81.2 | 81 | 486 | Alpha-hemolysin OS=Staphylococcus aureus (strain NCTC 8325) GN=hly PE=1 SV=1 | | | 34.1107 | 33.938 |
| sp\|Q2FZU0\|G6PI_STAA8 | 83.1 | 71 | 442 | Glucose-6-phosphate isomerase OS=Staphylococcus aureus (strain NCTC 8325) GN=pgi PE=3 S | 33.5148 | 33.6797 | 32.4696 | 32.3625 |
| sp\|Q2G0N1\|EFG_STAA8 | 66.5 | 71 | 421 | Elongation factor G OS=Staphylococcus aureus (strain NCTC 8325) GN=fusA PE=1 SV=3 | 33.6493 | 33.9187 | 30.1219 | 30.1163 |
| tr\|Q2G2A3\|Q2G2A3_STAA8 | 82.5 | 60 | 413 | Dihydrolipoyl dehydrogenase OS=Staphylococcus aureus (strain NCTC 8325) GN=SAOUHSC_0: | 34.0858 | 34.3673 | 32.5149 | 32.366 |
| sp\|Q2G093\|LTAS_STAA8 | 56.3 | 74 | 392 | Lipoteichoic acid synthase OS=Staphylococcus aureus (strain NCTC 8325) GN=ltaS PE=1 SV=1 | 31.6081 | 31.9913 | 32.8179 | 32.7488 |
| sp\|Q2FYU7\|CATA_STAA8 | 76.4 | 64 | 357 | Catalase OS=Staphylococcus aureus (strain NCTC 8325) GN=katA PE=2 SV=2 | 30.6605 | 31.1026 | 32.599 | 32.4084 |
| tr\|Q2FY60\|Q2FY60_STAA8 | 85.0 | 61 | 344 | 6-phosphogluconate dehydrogenase, decarboxylating OS=Staphylococcus aureus (strain NCTC | 32.7907 | 33.0608 | 32.0267 | 31.7308 |
| tr\|Q2G1U3\|Q2G1U3_STAA8 | 67.4 | 67 | 323 | Oligoendopeptidase F OS=Staphylococcus aureus (strain NCTC 8325) GN=SAOUHSC_00937 PE | 31.9436 | 32.3443 | 31.4839 | 31.7369 |
| sp\|Q2G031\|PGK_STAA8 | 82.1 | 59 | 310 | Phosphoglycerate kinase OS=Staphylococcus aureus (strain NCTC 8325) GN=pgk PE=3 SV=1 | 33.2785 | 33.5431 | 30.9573 | 31.1084 |
| sp\|P0A0B7\|AHPC_STAA8 | 74.6 | 30 | 305 | Alkyl hydroperoxide reductase subunit C OS=Staphylococcus aureus (strain NCTC 8325) GN=al | 33.2178 | 33.5135 | 32.8337 | 32.784 |
| tr\|Q2G2A5\|Q2G2A5_STAA8 | 98.8 | 59 | 303 | Pyruvate dehydrogenase complex, E1 component, pyruvate dehydrogenase beta subunit, put | 33.3578 | 33.6043 | 31.5368 | 31.4973 |
| tr\|Q2FWD3\|Q2FWD3_STAA8 | 94.1 | 53 | 299 | Uncharacterized protein OS=Staphylococcus aureus (strain NCTC 8325) GN=SAOUHSC_02366 | 33.3951 | 33.6854 | 31.5106 | 31.3109 |
| sp\|Q2FV52\|ISAA_STAA8 | 75.1 | 35 | 295 | Probable transglycosylase IsaA OS=Staphylococcus aureus (strain NCTC 8325) GN=isaA PE=1 S | 32.7104 | 33.0608 | 31.9539 | 31.8456 |
| sp\|Q2FWV5\|CHIPS_STAA8 | 76.5 | 52 | 287 | Chemotaxis inhibitory protein OS=Staphylococcus aureus (strain NCTC 8325) GN=chp PE=1 SV | 34.4721 | 34.8722 | | |
| sp\|P00766\|CTRA_BOVIN | 78.0 | 31 | 285 | Chymotrypsinogen A | 34.5879 | 34.9325 | 33.6598 | 33.8561 |
| sp\|Q2FZ23\|EFTS_STAA8 | 80.9 | 43 | 269 | Elongation factor Ts OS=Staphylococcus aureus (strain NCTC 8325) GN=tsf PE=3 SV=1 | 32.2993 | 32.7037 | 30.9299 | 30.8809 |
| tr\|Q2G010\|Q2G010_STAA8 | 63.6 | 38 | 267 | Thermonuclease OS=Staphylococcus aureus (strain NCTC 8325) GN=SAOUHSC_00818 PE=4 SV | 28.6875 | 29.0504 | 33.0955 | 33.0552 |
| tr\|Q2G170\|Q2G170_STAA8 | 77.0 | 46 | 261 | 5'-nucleotidase, lipoprotein e(P4) family OS=Staphylococcus aureus (strain NCTC 8325) GN=S/ | 32.3548 | 32.502 | 29.1227 | 29.3615 |
| sp\|P0A0J3\|SODM1_STAA8 | 97.0 | 40 | 259 | Superoxide dismutase [Mn] 1 OS=Staphylococcus aureus (strain NCTC 8325) GN=sodA PE=1 S\ | 33.576 | 34.0043 | 32.1479 | 31.8921 |
| contam_sp\|P02533\|K1CN_HUMAN | 62.8 | 40 | 245 | Keratin, type I cytoskeletal 14 (Cytokeratin 14) (K14) (CK 14) - Homo sapiens (Human). MW: 5: | 32.1276 | 32.4139 | 31.9286 | 31.7465 |
| tr\|Q2G2A4\|Q2G2A4_STAA8 | 56.3 | 39 | 228 | Dihydrolipoamide acetyltransferase component of pyruvate dehydrogenase complex OS=Stap | 32.5396 | 32.7169 | 30.218 | 30.2946 |
| sp\|Q2FZ82\|SYI_STAA8 | 63.9 | 70 | 224 | Isoleucine--tRNA ligase OS=Staphylococcus aureus (strain NCTC 8325) GN=ileS PE=3 SV=1 | 32.0205 | 32.3409 | 29.8388 | 29.7955 |
| contam_sp\|P13647\|K2C5_HUMAN | 47.8 | 44 | 221 | Keratin, type II cytoskeletal 5 (Cytokeratin 5) (K5) (CK 5) (58 kDa cytokeratin) - Homo sapiens ( | 32.4453 | 32.7011 | 31.4794 | 32.1157 |
| tr\|Q2G2C1\|Q2G2C1_STAA8 | 58.9 | 75 | 221 | Pyruvate carboxylase OS=Staphylococcus aureus (strain NCTC 8325) GN=SAOUHSC_01064 PE= | 31.4184 | 31.71 | 30.1564 | 30.7965 |
| tr\|Q2FUX4\|Q2FUX4_STAA8 | 61.0 | 56 | 220 | Aureolysin, putative OS=Staphylococcus aureus (strain NCTC 8325) GN=SAOUHSC_02971 PE=4 | 25.4706 | 23.327 | 33.453 | 33.4324 |
| tr\|Q2FWB8\|Q2FWB8_STAA8 | 89.4 | 42 | 219 | Purine nucleoside phosphorylase DeoD-type OS=Staphylococcus aureus (strain NCTC 8325) GI | 33.3985 | 33.6702 | 31.1977 | 30.9565 |
| sp\|Q2G218\|LDH1_STAA8 | 77.3 | 38 | 219 | L-lactate dehydrogenase 1 OS=Staphylococcus aureus (strain NCTC 8325) GN=ldh1 PE=3 SV=2 | 32.8077 | 33.2176 | 31.3412 | 31.101 |
| sp\|Q2FV17\|ALF1_STAA8 | 83.1 | 49 | 218 | Fructose-bisphosphate aldolase class 1 OS=Staphylococcus aureus (strain NCTC 8325) GN=fda | 32.6528 | 32.8561 | 30.923 | 31.5577 |
| tr\|Q2G0J0\|Q2G0J0_STAA8 | 87.8 | 48 | 207 | Phosphate acetyltransferase OS=Staphylococcus aureus (strain NCTC 8325) GN=SAOUHSC_00: | 32.0939 | 31.9862 | 32.0531 | 31.9769 |
| sp\|Q2G1Y5\|LDH2_STAA8 | 68.7 | 37 | 199 | L-lactate dehydrogenase 2 OS=Staphylococcus aureus (strain NCTC 8325) GN=ldh2 PE=3 SV=1 | 31.6183 | 31.8293 | 31.9286 | 31.8807 |
| tr\|Q2FYG2\|Q2FYG2_STAA8 | 93.3 | 27 | 198 | DNA-binding protein HU, putative OS=Staphylococcus aureus (strain NCTC 8325) GN=SAOUHS | 33.8636 | 34.0019 | 30.67 | 30.5239 |
| sp\|Q2G030\|TPIS_STAA8 | 78.7 | 35 | 197 | Triosephosphate isomerase OS=Staphylococcus aureus (strain NCTC 8325) GN=tpiA PE=3 SV=: | 32.5679 | 33.0073 | 31.0169 | 30.8653 |
| tr\|Q2FYY6\|Q2FYY6_STAA8 | 65.9 | 45 | 197 | Glutamine synthetase OS=Staphylococcus aureus (strain NCTC 8325) GN=SAOUHSC_01287 PE | 31.8452 | 32.2882 | 31.3362 | 31.3237 |
| sp\|Q2G296\|FTHS_STAA8 | 65.2 | 40 | 197 | Formate--tetrahydrofolate ligase OS=Staphylococcus aureus (strain NCTC 8325) GN=fhs PE=3 | 31.2928 | 31.1307 | 31.5194 | 31.4762 |
| sp\|Q2FZT4\|Y906_STAA8 | 81.7 | 37 | 194 | Uncharacterized protein SAOUHSC_00906 OS=Staphylococcus aureus (strain NCTC 8325) GN= | 31.7405 | 31.883 | 30.728 | 30.8912 |
| contam_sp\|P48668\|K2CE_HUMAN | 48.8 | 35 | 183 | Keratin, type II cytoskeletal 6E (Cytokeratin 6E) (CK 6E) (K6e keratin) - Homo sapiens (Human). | 32.43 | 32.7502 | 31.7766 | 32.0972 |
| sp\|Q2G155\|LIP2_STAA8 | 52.3 | 48 | 181 | Lipase 2 OS=Staphylococcus aureus (strain NCTC 8325) GN=lip2 PE=1 SV=1 | | | 32.4759 | 32.4679 |
| sp\|Q2G222\|Y2979_STAA8 | 55.3 | 47 | 176 | N-acetylmuramoyl-L-alanine amidase domain-containing protein SAOUHSC_02979 OS=Staphy | 23.7304 | 23.9202 | 31.8599 | 31.6206 |
| tr\|Q2G029\|Q2G029_STAA8 | 75.6 | 45 | 173 | 2,3-bisphosphoglycerate-independent phosphoglycerate mutase OS=Staphylococcus aureus ( | 31.015 | 31.2563 | 29.592 | 29.5232 |
| tr\|Q2FY66\|Q2FY66_STAA8 | 58.9 | 46 | 172 | Glucose-6-phosphate 1-dehydrogenase OS=Staphylococcus aureus (strain NCTC 8325) GN=zw | 30.5753 | 30.8135 | 30.322 | 30.2363 |
| sp\|Q2G241\|SYE_STAA8 | 63.6 | 37 | 164 | Glutamate--tRNA ligase OS=Staphylococcus aureus (strain NCTC 8325) GN=gltX PE=3 SV=1 | 30.7327 | 30.4601 | 30.4981 | 30.5532 |
| tr\|Q2FZG4\|Q2FZG4_STAA8 | 62.2 | 29 | 161 | Pyruvate dehydrogenase complex, E1 component, alpha subunit, putative OS=Staphylococcus | 31.8628 | 32.2059 | 30.0996 | 29.634 |
| tr\|Q2G0X3\|Q2G0X3_STAA8 | 29.9 | 27 | 159 | Uncharacterized protein OS=Staphylococcus aureus (strain NCTC 8325) GN=SAOUHSC_00400 | 32.499 | 32.7655 | 28.2161 | 27.5229 |
| sp\|Q2G0Y6\|GUAA_STAA8 | 62.0 | 47 | 157 | GMP synthase [glutamine-hydrolyzing] OS=Staphylococcus aureus (strain NCTC 8325) GN=gua | 31.5875 | 31.6781 | 28.5371 | 28.8261 |
| sp\|Q2FZK7\|ATL_STAA8 | 40.1 | 54 | 150 | Bifunctional autolysin OS=Staphylococcus aureus (strain NCTC 8325) GN=atl PE=1 SV=1 | 28.4205 | 28.4986 | 30.4588 | 30.1626 |
| sp\|Q2FXB0\|LUKEV_STAA8 | 68.6 | 46 | 149 | Leucotoxin LukEv OS=Staphylococcus aureus (strain NCTC 8325) GN=lukEv PE=1 SV=2 | | | 32.5129 | 32.2187 |
| sp\|Q2G0L5\|SDRC_STAA8 | 28.2 | 38 | 137 | Serine-aspartate repeat-containing protein C OS=Staphylococcus aureus (strain NCTC 8325) G | 26.4967 | 26.796 | 31.7549 | 31.7531 |
| sp\|Q2FXQ6\|TIG_STAA8 | 58.9 | 33 | 132 | Trigger factor OS=Staphylococcus aureus (strain NCTC 8325) GN=tig PE=3 SV=1 | 31.6029 | 31.7804 | 29.1886 | 29.4793 |
| sp\|Q2FXA0\|Y1977_STAA8 | 94.7 | 26 | 127 | UPF0342 protein SAOUHSC_01977 OS=Staphylococcus aureus (strain NCTC 8325) GN=SAOUH | 32.252 | 32.5086 | 29.7186 | 29.7055 |
| tr\|Q2G041\|Q2G041_STAA8 | 74.3 | 30 | 127 | Thioredoxin reductase OS=Staphylococcus aureus (strain NCTC 8325) GN=SAOUHSC_00785 PE | 30.7762 | 30.7386 | 30.6443 | 30.6565 |
| sp\|Q2FXC5\|SPLD_STAA8 | 79.1 | 34 | 127 | Serine protease SplD OS=Staphylococcus aureus (strain NCTC 8325) GN=splD PE=1 SV=1 | | | 31.8189 | 31.5261 |
| sp\|Q2FUX7\|ARCA_STAA8 | 58.4 | 39 | 126 | Arginine deiminase OS=Staphylococcus aureus (strain NCTC 8325) GN=arcA PE=3 SV=1 | 31.9724 | 32.2109 | 23.5525 | 25.9061 |
| tr\|Q2FXE8\|Q2FXE8_STAA8 | 76.4 | 27 | 124 | Transaldolase OS=Staphylococcus aureus (strain NCTC 8325) GN=SAOUHSC_01901 PE=4 SV=1 | 31.4915 | 31.8751 | 30.9775 | 30.5295 |
| sp\|Q2FXH9\|PEPVL_STAA8 | 44.3 | 29 | 124 | Putative dipeptidase SAOUHSC_01868 OS=Staphylococcus aureus (strain NCTC 8325) GN=SAC | 30.0507 | 30.0093 | 29.7008 | 30.0117 |
| sp\|O52582\|CDR_STAA8 | 68.7 | 39 | 124 | Coenzyme A disulfide reductase OS=Staphylococcus aureus (strain NCTC 8325) GN=cdr PE=1 S | 29.2397 | 29.7107 | 30.089 | 30.6281 |
| sp\|Q2FVK2\|HLGC_STAA8 | 61.0 | 31 | 121 | Gamma-hemolysin component C OS=Staphylococcus aureus (strain NCTC 8325) GN=hlgC PE=1 SV=1 | | | 31.6798 | 31.258 |
| sp\|Q2FVK5\|SBI_STAA8 | 50.0 | 31 | 117 | Immunoglobulin-binding protein sbi OS=Staphylococcus aureus (strain NCTC 8325) GN=sbi PE | 29.8498 | 29.9908 | 30.3549 | 30.1264 |
| contam_tr\|Q61869 | 16.1 | 20 | 115 | Keratin 2 epidermis - Mus musculus (Mouse). MW: 70977.30 pI: 8.23 | 31.3657 | 31.7489 | 31.4427 | 31.5728 |
| tr\|Q2FUX8\|Q2FUX8_STAA8 | 58.6 | 32 | 114 | Ornithine carbamoyltransferase OS=Staphylococcus aureus (strain NCTC 8325) GN=argF PE=3 | 32.2209 | 32.2211 | 27.4638 | 27.4922 |
| sp\|P95689\|SYS_STAA8 | 62.1 | 35 | 114 | Serine--tRNA ligase OS=Staphylococcus aureus (strain NCTC 8325) GN=serS PE=3 SV=1 | 30.9623 | 31.3931 | 28.1989 | 28.2331 |
| sp\|Q2FXZ2\|DNAK_STAA8 | 63.8 | 31 | 114 | Chaperone protein DnaK OS=Staphylococcus aureus (strain NCTC 8325) GN=dnaK PE=3 SV=1 | 29.8498 | 30.3161 | 29.4668 | 29.1325 |
| sp\|Q2FXC8\|SPLF_STAA8 | 65.3 | 30 | 114 | Serine protease SplF OS=Staphylococcus aureus (strain NCTC 8325) GN=splF PE=1 SV=1 | | | 31.8189 | 31.4365 |
| sp\|Q2G0F8\|SYR_STAA8 | 58.4 | 39 | 112 | Arginine--tRNA ligase OS=Staphylococcus aureus (strain NCTC 8325) GN=argS PE=3 SV=1 | 30.6042 | 30.6614 | 28.0327 | 28.0334 |
| sp\|Q2FVK8\|GPMA_STAA8 | 65.8 | 26 | 112 | 2,3-bisphosphoglycerate-dependent phosphoglycerate mutase OS=Staphylococcus aureus (stı | 30.3789 | 30.2677 | 29.5763 | 29.4072 |
| sp\|Q2FWM8\|HLD_STAA8 | 92.3 | 9 | 112 | Delta-hemolysin OS=Staphylococcus aureus (strain NCTC 8325) GN=hld PE=3 SV=3 | | | 35.3477 | 35.1362 |
| sp\|Q2FWV6\|SCIN_STAA8 | 60.3 | 21 | 110 | Staphylococcal complement inhibitor OS=Staphylococcus aureus (strain NCTC 8325) GN=scn F | 33.9843 | 34.2551 | 29.5008 | 29.3989 |
| tr\|Q2FZH5\|Q2FZH5_STAA8 | 45.1 | 30 | 108 | Phosphoenolpyruvate-protein phosphotransferase OS=Staphylococcus aureus (strain NCTC 83 | 31.5508 | 31.3315 | 30.2768 | 30.0554 |
| tr\|Q2G2P2\|Q2G2P2_STAA8 | 63.5 | 30 | 104 | Globin domain protein OS=Staphylococcus aureus (strain NCTC 8325) GN=SAOUHSC_00204 P | 31.0443 | 31.66 | 25.7848 | 25.4522 |
| sp\|Q2FWN9\|LUKL2_STAA8 | 50.1 | 31 | 104 | Uncharacterized leukocidin-like protein 2 OS=Staphylococcus aureus (strain NCTC 8325) GN=SAOUHSC_02243 PE=: | | | 31.3412 | 31.2588 |
| sp\|Q2FXR8\|SYV_STAA8 | 40.5 | 40 | 103 | Valine--tRNA ligase OS=Staphylococcus aureus (strain NCTC 8325) GN=valS PE=3 SV=1 | 30.3343 | 30.7376 | 27.7953 | 28.0964 |
| tr\|Q2FW27\|Q2FW27_STAA8 | 74.9 | 20 | 102 | Adenylate kinase OS=Staphylococcus aureus (strain NCTC 8325) GN=adk PE=3 SV=1 | 29.5106 | 29.8844 | 29.2098 | 29.3248 |
| sp\|Q2FXC3\|SPLB_STAA8 | 66.3 | 23 | 102 | Serine protease SplB OS=Staphylococcus aureus (strain NCTC 8325) GN=splB PE=1 SV=1 | | | 32.2412 | 32.3596 |
| sp\|Q2G0J1\|Y573_STAA8 | 56.4 | 19 | 101 | Putative heme-dependent peroxidase SAOUHSC_00573 OS=Staphylococcus aureus (strain NC | 30.0641 | 29.864 | 29.9938 | 29.8332 |
| tr\|Q2G1W1\|Q2G1W1_STAA8 | 63.3 | 19 | 99 | Secretory antigen SsaA, putative OS=Staphylococcus aureus (strain NCTC 8325) GN=SAOUHSC | 28.3637 | 29.5329 | 30.4786 | 30.2803 |
| sp\|Q2FWZ8\|FTN_STAA8 | 68.1 | 21 | 98 | Bacterial non-heme ferritin OS=Staphylococcus aureus (strain NCTC 8325) GN=ftnA PE=1 SV=1 | 30.5158 | 30.7827 | 30.8588 | 30.7425 |
| tr\|Q2G1R9\|Q2G1R9_STAA8 | 50.7 | 34 | 97 | Methionine--tRNA ligase OS=Staphylococcus aureus (strain NCTC 8325) GN=metG PE=3 SV=1 | 30.4115 | 30.4859 | 28.32 | 29.0016 |
| sp\|Q2G0G1\|ADH_STAA8 | 57.4 | 20 | 96 | Alcohol dehydrogenase OS=Staphylococcus aureus (strain NCTC 8325) GN=adh PE=3 SV=1 | 29.865 | 30.3423 | 30.5362 | 30.4681 |
| sp\|Q2FV21\|PANB_STAA8 | 72.1 | 22 | 94 | 3-methyl-2-oxobutanoate hydroxymethyltransferase OS=Staphylococcus aureus (strain NCTC | 30.2171 | 29.9979 | 29.3981 | 29.3678 |

| | | | | | | | | |
|---|---|---|---|---|---|---|---|---|
| sp\|Q2G036\|CLPP_STAA8 | 50.8 | 21 | 93 | ATP-dependent Clp protease proteolytic subunit OS=Staphylococcus aureus (strain NCTC 832! | 30.7847 | 31.1228 | 28.546 | 28.6913 |
| sp\|Q2FVK1\|HLGB_STAA8 | 51.7 | 32 | 93 | Gamma-hemolysin component B OS=Staphylococcus aureus (strain NCTC 8325) GN=hlgB PE=1 SV=1 | | | 31.2899 | 31.1914 |
| sp\|Q2G2S0\|PUR8_STAA8 | 49.7 | 26 | 91 | Adenylosuccinate lyase OS=Staphylococcus aureus (strain NCTC 8325) GN=purB PE=3 SV=1 | 29.6289 | 30.165 | 28.9219 | 28.8936 |
| tr\|Q2FWB7\|Q2FWB7_STAA8 | 78.2 | 23 | 90 | Uncharacterized protein OS=Staphylococcus aureus (strain NCTC 8325) GN=SAOUHSC_02381 | 32.0126 | 32.2238 | 28.912 | 28.6659 |
| sp\|Q2G0N0\|EFTU_STAA8 | 71.8 | 26 | 90 | Elongation factor Tu OS=Staphylococcus aureus (strain NCTC 8325) GN=tuf PE=3 SV=1 | 30.7932 | 31.1066 | 29.0017 | 28.9998 |
| sp\|Q2G0L4\|SDRD_STAA8 | 28.8 | 29 | 89 | Serine-aspartate repeat-containing protein D OS=Staphylococcus aureus (strain NCTC 8325) G | 26.7996 | 27.0301 | 30.8141 | 30.7815 |
| sp\|Q2FZ20\|PNP_STAA8 | 48.3 | 37 | 87 | Polyribonucleotide nucleotidyltransferase OS=Staphylococcus aureus (strain NCTC 8325) GN= | 29.535 | 30.3358 | 27.7305 | 26.9468 |
| tr\|Q2G0M4\|Q2G0M4_STAA8 | 61.2 | 25 | 86 | Branched-chain-amino-acid aminotransferase OS=Staphylococcus aureus (strain NCTC 8325) ( | 29.299 | 29.547 | 30.3657 | 30.2473 |
| sp\|Q2G122\|METE_STAA8 | 44.5 | 34 | 86 | 5-methyltetrahydropteroyltriglutamate--homocysteine methyltransferase OS=Staphylococcus | 27.4941 | 27.4743 | 30.6004 | 29.5476 |
| tr\|Q2FZD8\|Q2FZD8_STAA8 | 34.4 | 27 | 85 | Phenylalanine--tRNA ligase beta subunit OS=Staphylococcus aureus (strain NCTC 8325) GN=pł | 28.8177 | 29.141 | 29.5623 | 29.4646 |
| sp\|Q59801\|HYSA_STAA8 | 20.4 | 26 | 84 | Hyaluronate lyase OS=Staphylococcus aureus (strain NCTC 8325) GN=hysA PE=3 SV=1 | | | 30.728 | 30.6387 |
| contam_tr\|Q9EQD6,contam_tr\|Q9EQD7 | 23.7 | 15 | 82 | Keratin intermediate filament 16b - Mus musculus (Mouse). MW: 51965.95 pI: 5.13 | 31.2098 | 31.3866 | 30.6357 | 30.2787 |
| sp\|Q2FWE5\|GLYA_STAA8 | 50.0 | 23 | 81 | Serine hydroxymethyltransferase OS=Staphylococcus aureus (strain NCTC 8325) GN=glyA PE=3 | 29.3706 | 28.8923 | 29.9678 | 29.7809 |
| sp\|Q2G2R8\|SSPP_STAA8 | 44.1 | 28 | 81 | Staphopain A OS=Staphylococcus aureus (strain NCTC 8325) GN=sspP PE=1 SV=1 | 25.5483 | 24.6888 | 31.1044 | 30.9905 |
| contam_sp\|P05787\|K2C8_HUMAN | 7.3 | 9 | 80 | Keratin, type II cytoskeletal 8 (Cytokeratin 8) (K8) (CK 8) - Homo sapiens (Human). MW: 53543 | 30.5158 | 30.8024 | 30.7199 | 30.7523 |
| contam_sp\|P19012\|K1CO_HUMAN | 12.7 | 10 | 78 | Keratin, type I cytoskeletal 15 (Cytokeratin 15) (K15) (CK 15) - Homo sapiens (Human). MW: 4! | 31.037 | 31.3672 | 30.728 | 30.4951 |
| sp\|Q2G227\|DEOB_STAA8 | 55.6 | 21 | 76 | Phosphopentomutase OS=Staphylococcus aureus (strain NCTC 8325) GN=deoB PE=3 SV=1 | 29.7009 | 30.2069 | 27.3122 | 26.4405 |
| sp\|Q2FXH2\|SYL_STAA8 | 33.7 | 27 | 76 | Leucine--tRNA ligase OS=Staphylococcus aureus (strain NCTC 8325) GN=leuS PE=3 SV=1 | 28.8148 | 29.817 | 27.2291 | 27.7563 |
| tr\|Q2FVA3\|Q2FVA3_STAA8 | 62.5 | 23 | 75 | D-lactate dehydrogenase, putative OS=Staphylococcus aureus (strain NCTC 8325) GN=SAOUH | 30.4852 | 31.0132 | | |
| tr\|Q2G077\|Q2G077_STAA8 | 71.2 | 30 | 72 | Ribonucleotide-disphosphate reductase beta chain, putative OS=Staphylococcus aureus (strai | 30.1167 | 30.2716 | | 24.6744 |
| tr\|Q2FYS9\|Q2FYS9_STAA8 | 36.1 | 26 | 71 | Aconitate hydratase OS=Staphylococcus aureus (strain NCTC 8325) GN=SAOUHSC_01347 PE=3 | 27.1309 | 27.399 | 29.3844 | 29.7511 |
| sp\|Q2FZW6\|DLTA_STAA8 | 42.1 | 24 | 70 | D-alanine--poly(phosphoribitol) ligase subunit 1 OS=Staphylococcus aureus (strain NCTC 8325 | 29.2464 | 29.1215 | 27.1204 | 26.8414 |
| sp\|Q2G261\|SODM2_STAA8 | 73.4 | 19 | 70 | Superoxide dismutase [Mn/Fe] 2 OS=Staphylococcus aureus (strain NCTC 8325) GN=sodM PE= | 29.1735 | 29.4385 | 30.3657 | 30.3366 |
| sp\|Q2FXG2\|RISB_STAA8 | 67.5 | 17 | 69 | 6,7-dimethyl-8-ribityllumazine synthase OS=Staphylococcus aureus (strain NCTC 8325) GN=rib | 30.3115 | 30.1671 | 30.1206 | 30.3611 |
| tr\|Q2G248\|Q2G248_STAA8 | 35.8 | 17 | 69 | Uncharacterized protein OS=Staphylococcus aureus (strain NCTC 8325) GN=SAOUHSC_01852 | 28.0726 | 28.216 | 29.3352 | 29.4298 |
| sp\|Q2FV67\|ROCA_STAA8 | 49.2 | 26 | 68 | 1-pyrroline-5-carboxylate dehydrogenase OS=Staphylococcus aureus (strain NCTC 8325) GN=r | 29.314 | 29.7081 | 28.4211 | 28.8379 |
| sp\|Q2G1D8\|PFLB_STAA8 | 35.1 | 23 | 68 | Formate acetyltransferase OS=Staphylococcus aureus (strain NCTC 8325) GN=pflB PE=3 SV=1 | 28.7735 | 29.1415 | 28.5893 | 28.4589 |
| sp\|Q2FVW4\|Y2577_STAA8 | 63.7 | 21 | 67 | Putative 2-hydroxyacid dehydrogenase SAOUHSC_02577 OS=Staphylococcus aureus (strain N( | 29.9818 | 30.2127 | 28.8333 | 28.125 |
| tr\|Q2G2G0\|Q2G2G0_STAA8 | 52.1 | 14 | 66 | Uncharacterized protein OS=Staphylococcus aureus (strain NCTC 8325) GN=SAOUHSC_00717 | 28.728 | 29.7036 | 29.2614 | 29.5143 |
| contam_sp\|P08727\|K1CS_HUMAN | 17.3 | 12 | 64 | Keratin, type I cytoskeletal 19 (Cytokeratin 19) (K19) (CK 19) - Homo sapiens (Human). MW: 4 | 30.7502 | 31.2172 | 30.6785 | 30.4372 |
| tr\|Q2FY40\|Q2FY40_STAA8 | 54.4 | 22 | 64 | Proline dipeptidase, putative OS=Staphylococcus aureus (strain NCTC 8325) GN=SAOUHSC_01 | 29.2375 | 29.132 | 28.5978 | 28.875 |
| tr\|Q2G0F2\|Q2G0F2_STAA8 | 63.7 | 19 | 64 | Uncharacterized protein OS=Staphylococcus aureus (strain NCTC 8325) GN=SAOUHSC_00617 | 29.1898 | 29.9744 | 28.8851 | 28.7025 |
| sp\|Q2G2Q1\|RS15_STAA8 | 71.9 | 11 | 63 | 30S ribosomal protein S15 OS=Staphylococcus aureus (strain NCTC 8325) GN=rpsO PE=1 SV=1 | 30.9851 | 30.8149 | | |
| sp\|Q2FXB1\|LUKDV_STAA8 | 59.3 | 25 | 63 | Leucotoxin LukDv OS=Staphylococcus aureus (strain NCTC 8325) GN=lukDv PE=1 SV=1 | | | 30.8948 | 30.5489 |
| tr\|Q2FZU5\|Q2FZU5_STAA8 | 42.5 | 19 | 62 | Glutamate dehydrogenase OS=Staphylococcus aureus (strain NCTC 8325) GN=SAOUHSC_0089 | 28.7187 | 28.8707 | 29.6134 | 28.9215 |
| sp\|Q2FWY1\|PPAC_STAA8 | 59.5 | 24 | 60 | Probable manganese-dependent inorganic pyrophosphatase OS=Staphylococcus aureus (strai | 30.0507 | 30.5311 | 28.0905 | 24.5016 |
| sp\|Q2FYG7\|NDK_STAA8 | 73.8 | 13 | 60 | Nucleoside diphosphate kinase OS=Staphylococcus aureus (strain NCTC 8325) GN=ndk PE=3 S | 29.5479 | 29.7987 | 28.6021 | 28.5982 |
| sp\|Q2FWP0\|LUKL1_STAA8 | 48.2 | 19 | 60 | Uncharacterized leukocidin-like protein 1 OS=Staphylococcus aureus (strain NCTC 8325) GN=SAOUHSC_02241 PE=: | | | 29.9049 | 32.1094 |
| sp\|Q2G0B1\|MGRA_STAA8 | 67.3 | 15 | 56 | HTH-type transcriptional regulator MgrA OS=Staphylococcus aureus (strain NCTC 8325) GN=m | 28.461 | 28.7797 | 28.7756 | 28.8811 |
| sp\|Q2G0K7\|HPS_STAA8 | 67.1 | 15 | 55 | 3-hexulose-6-phosphate synthase OS=Staphylococcus aureus (strain NCTC 8325) GN=SAOUHS | 29.8189 | 30.1811 | 27.9444 | 28.2463 |
| tr\|Q2FZB0\|Q2FZB0_STAA8 | 48.0 | 17 | 55 | Ornithine carbamoyltransferase OS=Staphylococcus aureus (strain NCTC 8325) GN=argF PE=3 | 29.7521 | 29.88 | 25.5134 | 26.0248 |
| tr\|Q2G253\|Q2G253_STAA8 | 28.8 | 21 | 55 | Uncharacterized protein OS=Staphylococcus aureus (strain NCTC 8325) GN=SAOUHSC_00025 | 22.3732 | 21.9259 | 29.1085 | 29.4256 |
| sp\|Q2FXR3\|HEM2_STAA8 | 50.0 | 18 | 54 | Delta-aminolevulinic acid dehydratase OS=Staphylococcus aureus (strain NCTC 8325) GN=hen | 28.1046 | 28.9413 | 27.4841 | 27.0111 |
| tr\|Q2FX14\|Q2FX14_STAA8 | 49.3 | 21 | 53 | Aminopeptidase PepS, putative OS=Staphylococcus aureus (strain NCTC 8325) GN=SAOUHSC_ | 28.4242 | 28.7419 | 28.0261 | 27.8499 |
| sp\|Q2G2J2\|SSAA2_STAA8 | 50.6 | 14 | 53 | Staphylococcal secretory antigen ssaA2 OS=Staphylococcus aureus (strain NCTC 8325) GN=ssa | 27.9325 | 27.4915 | 29.3471 | 29.2046 |
| contam_gi\|136429\|sp\|P00761\|TRYP_PIG | 34.2 | 8 | 52 | Trypsin precursor MW: 24409.68 pI: 7.00 | 30.9926 | 31.1456 | 31.515 | 31.2556 |
| contam_sp\|P13646\|K1CM_HUMAN | 11.4 | 8 | 52 | Keratin, type I cytoskeletal 13 (Cytokeratin 13) (K13) (CK 13) - Homo sapiens (Human). MW: 4! | 29.865 | 29.9438 | 30.3109 | 29.9715 |
| tr\|Q2FYQ2\|Q2FYQ2_STAA8 | 33.3 | 20 | 52 | Uncharacterized protein OS=Staphylococcus aureus (strain NCTC 8325) GN=SAOUHSC_01383 | 28.9542 | 28.9183 | 28.0129 | 27.9628 |
| tr\|Q2FZY6\|Q2FZY6_STAA8 | 49.9 | 21 | 52 | Uncharacterized protein OS=Staphylococcus aureus (strain NCTC 8325) GN=SAOUHSC_00848 | 28.7311 | 28.8176 | 27.131 | 27.1853 |
| sp\|Q2FZS0\|FABH_STAA8 | 36.7 | 15 | 51 | 3-oxoacyl-[acyl-carrier-protein] synthase 3 OS=Staphylococcus aureus (strain NCTC 8325) GN= | 29.9244 | 28.5729 | 27.8645 | 29.7689 |
| tr\|Q2G1H0\|Q2G1H0_STAA8 | 40.1 | 21 | 51 | Indolepyruvate decarboxylase, putative OS=Staphylococcus aureus (strain NCTC 8325) GN=SA | 29.2352 | 29.2383 | 27.2654 | 26.5182 |
| tr\|Q2FWW1\|Q2FWW1_STAA8 | 28.1 | 19 | 51 | MHC class II analog protein OS=Staphylococcus aureus (strain NCTC 8325) GN=SAOUHSC_021 | 25.7393 | 25.8867 | 30.3974 | 30.0184 |
| sp\|P60430\|RL2_STAA8 | 41.2 | 12 | 50 | 50S ribosomal protein L2 OS=Staphylococcus aureus (strain NCTC 8325) GN=rplB PE=1 SV=1 | 30.4645 | 30.5919 | 24.5094 | 26.2114 |
| sp\|Q2FW81\|URTF_STAA8 | 39.7 | 19 | 50 | Probable uridylyltransferase SAOUHSC_02423 OS=Staphylococcus aureus (strain NCTC 8325) ( | 28.2226 | 28.4937 | 28.4873 | 26.6667 |
| sp\|Q2FUU5\|LIP1_STAA8 | 29.1 | 22 | 50 | Lipase 1 OS=Staphylococcus aureus (strain NCTC 8325) GN=lipA PE=1 SV=1 | | | 29.8436 | 29.9515 |
| sp\|Q2FW32\|RPOA_STAA8 | 53.8 | 20 | 49 | DNA-directed RNA polymerase subunit alpha OS=Staphylococcus aureus (strain NCTC 8325) G | 28.9408 | 29.2467 | 27.5335 | 26.8459 |
| sp\|Q2FXM8\|PFKA_STAA8 | 46.9 | 19 | 48 | ATP-dependent 6-phosphofructokinase OS=Staphylococcus aureus (strain NCTC 8325) GN=pfk | 28.9542 | 28.7134 | 26.818 | 23.8988 |
| tr\|Q2FVL2\|Q2FVL2_STAA8 | 42.9 | 15 | 48 | Uncharacterized protein OS=Staphylococcus aureus (strain NCTC 8325) GN=SAOUHSC_02699 | 28.5215 | 27.8469 | 28.0905 | 27.7863 |
| sp\|Q2G2S6\|PRSA_STAA8 | 34.7 | 16 | 47 | Foldase protein PrsA OS=Staphylococcus aureus (strain NCTC 8325) GN=prsA PE=3 SV=1 | 23.1834 | 22.4796 | 29.9546 | 29.4775 |
| sp\|P48940\|RS7_STAA8 | 50.6 | 10 | 46 | 30S ribosomal protein S7 OS=Staphylococcus aureus (strain NCTC 8325) GN=rpsG PE=1 SV=2 | 29.4973 | 29.5407 | 27.401 | 27.3569 |
| tr\|Q2FVF9\|Q2FVF9_STAA8 | 41.9 | 13 | 46 | Uncharacterized protein OS=Staphylococcus aureus (strain NCTC 8325) GN=SAOUHSC_02755 | 29.2553 | 28.3775 | 29.4472 | 28.3512 |
| tr\|Q2FZ05\|Q2FZ05_STAA8 | 36.2 | 16 | 46 | Uncharacterized protein OS=Staphylococcus aureus (strain NCTC 8325) GN=SAOUHSC_01266 | 28.439 | 28.117 | 26.2943 | 26.4024 |
| sp\|P0C0V7\|GLMM_STAA8 | 35.7 | 18 | 45 | Phosphoglucosamine mutase OS=Staphylococcus aureus (strain NCTC 8325) GN=glmM PE=1 S | 29.3911 | 29.818 | 25.7271 | 25.318 |
| tr\|Q2G0Q8\|Q2G0Q8_STAA8 | 56.1 | 14 | 44 | Cysteine synthase OS=Staphylococcus aureus (strain NCTC 8325) GN=SAOUHSC_00488 PE=3 S | 29.4935 | 29.7728 | 26.8431 | 26.8563 |
| tr\|Q2FWR0\|Q2FWR0_STAA8 | 56.3 | 15 | 43 | Phi ETA orf 18-like protein OS=Staphylococcus aureus (strain NCTC 8325) GN=SAOUHSC_0222 | 29.0923 | 29.5289 | | |
| sp\|Q2FWD1\|PYRG_STAA8 | 28.0 | 17 | 43 | CTP synthase OS=Staphylococcus aureus (strain NCTC 8325) GN=pyrG PE=3 SV=1 | 28.8119 | 28.8451 | 26.9341 | 27.3484 |
| sp\|Q2G0M7\|HCHA_STAA8 | 41.8 | 16 | 43 | Protein deglycase HchA OS=Staphylococcus aureus (strain NCTC 8325) GN=hchA PE=3 SV=1 | 26.8793 | 27.4997 | 29.3256 | 29.1995 |
| sp\|Q2FZ37\|SUCC_STAA8 | 47.2 | 21 | 42 | Succinate--CoA ligase [ADP-forming] subunit beta OS=Staphylococcus aureus (strain NCTC 832 | 30.4749 | 28.4296 | 26.8112 | 26.1575 |
| sp\|Q2FXZ1\|GRPE_STAA8 | 86.5 | 15 | 42 | Protein GrpE OS=Staphylococcus aureus (strain NCTC 8325) GN=grpE PE=3 SV=1 | 29.6271 | 30.0649 | 23.8466 | 26.8663 |
| sp\|Q2G0Q1\|PDXS_STAA8 | 53.2 | 12 | 42 | Pyridoxal 5'-phosphate synthase subunit PdxS OS=Staphylococcus aureus (strain NCTC 8325) ( | 28.678 | 29.4912 | 25.8616 | 25.8773 |
| sp\|Q2FW30\|RS13_STAA8 | 57.9 | 11 | 42 | 30S ribosomal protein S13 OS=Staphylococcus aureus (strain NCTC 8325) GN=rpsM PE=1 SV=1 | 28.4464 | 28.7966 | 26.957 | 25.8097 |
| sp\|Q2FWX8\|Y2143_STAA8 | 47.1 | 17 | 41 | Uncharacterized protein SAOUHSC_02143 OS=Staphylococcus aureus (strain NCTC 8325) GN= | 30.4955 | 28.2452 | 28.0129 | 26.2796 |
| tr\|Q2FX74\|Q2FX74_STAA8 | 47.2 | 17 | 41 | Phage tail fiber protein, putative OS=Staphylococcus aureus (strain NCTC 8325) GN=SAOUHSC | 29.2838 | 29.5293 | | |
| sp\|Q2FZ51\|ACP_STAA8 | 62.3 | 6 | 41 | Acyl carrier protein OS=Staphylococcus aureus (strain NCTC 8325) GN=acpP PE=2 SV=1 | 29.2397 | 29.5559 | 29.1227 | 28.7416 |
| sp\|Q2FWN4\|CH60_STAA8 | 40.7 | 19 | 41 | 60 kDa chaperonin OS=Staphylococcus aureus (strain NCTC 8325) GN=groL PE=3 SV=1 | 28.5732 | 28.6738 | 24.9148 | |
| tr\|Q2FXI5\|Q2FXI5_STAA8 | 39.4 | 16 | 40 | Uncharacterized protein OS=Staphylococcus aureus (strain NCTC 8325) GN=SAOUHSC_01861 | 27.8549 | 29.0137 | 28.0062 | 27.5979 |
| tr\|Q2FZU3\|Q2FZU3_STAA8 | 48.9 | 15 | 40 | Uncharacterized protein OS=Staphylococcus aureus (strain NCTC 8325) GN=SAOUHSC_00897 | 23.2687 | 24.3087 | 29.5381 | 29.3237 |
| tr\|Q2FX43\|Q2FX43_STAA8 | 69.1 | 17 | 39 | DUTP pyrophosphatase OS=Staphylococcus aureus (strain NCTC 8325) GN=SAOUHSC_02057 F | 30.1296 | 30.1608 | | |
| sp\|Q2G0P0\|RL1_STAA8 | 66.5 | 16 | 39 | 50S ribosomal protein L1 OS=Staphylococcus aureus (strain NCTC 8325) GN=rplA PE=3 SV=1 | 28.9964 | 29.1528 | 25.1322 | 23.2635 |
| tr\|Q2G1W8\|Q2G1W8_STAA8 | 40.0 | 17 | 39 | Phage major capsid protein, HK97 family OS=Staphylococcus aureus (strain NCTC 8325) GN=S. | 28.6685 | 28.4174 | 27.5995 | 27.3067 |
| sp\|Q2FZY3\|Y851_STAA8 | 32.0 | 18 | 39 | UPF0051 protein SAOUHSC_00851 OS=Staphylococcus aureus (strain NCTC 8325) GN=SAOUH | 28.3942 | 29.0449 | 26.1524 | 25.3379 |
| tr\|Q2FZ74\|Q2FZ74_STAA8 | 25.5 | 10 | 39 | Dihydroorotase OS=Staphylococcus aureus (strain NCTC 8325) GN=pyrC PE=3 SV=1 | 28.0208 | 28.0851 | 26.4059 | 25.8048 |
| tr\|Q2FZS8\|Q2FZS8_STAA8 | 26.7 | 20 | 39 | Chaperone protein ClpB OS=Staphylococcus aureus (strain NCTC 8325) GN=clpB PE=3 SV=1 | 27.9625 | 28.62 | | 24.2026 |
| tr\|Q2G1I1\|Q2G1I1_STAA8 | 39.3 | 12 | 39 | Formate dehydrogenase OS=Staphylococcus aureus (strain NCTC 8325) GN=SAOUHSC_00142 | 26.1073 | 26.9669 | 29.1367 | 28.6392 |
| std_gi\|2190337\|gnl\|PID\|e321614 | 17.6 | 16 | 38 | (X58989) serum albumin [Bos taurus] gi\|3336842\|gnl\|PID\|e1311980 (Y17769) bovine serum | 28.6938 | 28.9976 | 27.2413 | 28.2398 |
| tr\|Q2FZQ7\|Q2FZQ7_STAA8 | 52.0 | 15 | 37 | Tryptophan--tRNA ligase OS=Staphylococcus aureus (strain NCTC 8325) GN=trpS PE=3 SV=1 | 28.4718 | 29.2051 | 25.3342 | 25.5574 |
| sp\|Q2FZU9\|PPI1_STAA8 | 51.8 | 8 | 37 | Putative peptidyl-prolyl cis-trans isomerase OS=Staphylococcus aureus (strain NCTC 8325) GN | 27.1309 | 27.6455 | 28.1398 | 27.9791 |
| tr\|Q2FX69\|Q2FX69_STAA8 | 30.8 | 21 | 36 | PhiETA ORF57-like protein OS=Staphylococcus aureus (strain NCTC 8325) GN=SAOUHSC_0202 | 29.4253 | 28.3694 | | |
| tr\|Q2FZV6\|Q2FZV6_STAA8 | 27.3 | 13 | 36 | Probable cytosol aminopeptidase OS=Staphylococcus aureus (strain NCTC 8325) GN=SAOUHS | 27.6698 | 27.9371 | 27.3462 | 27.7775 |
| sp\|Q2FZD9\|SYFA_STAA8 | 33.0 | 14 | 36 | Phenylalanine--tRNA ligase alpha subunit OS=Staphylococcus aureus (strain NCTC 8325) GN=ŗ | 26.889 | 27.8258 | 27.3122 | 27.4749 |

| | | | | | | | | |
|---|---|---|---|---|---|---|---|---|
| sp\|Q2G113\|RS6_STAA8 | 79.6 | 11 | 35 | 30S ribosomal protein S6 OS=Staphylococcus aureus (strain NCTC 8325) GN=rpsF PE=1 SV=1 | 29.0626 | 29.0884 | 26.9855 | 27.0888 |
| tr\|Q2G0X2\|Q2G0X2_STAA8 | 54.9 | 8 | 35 | Uncharacterized protein OS=Staphylococcus aureus (strain NCTC 8325) GN=SAOUHSC_00401 | 27.9575 | 27.3994 | 29.5743 | 29.2361 |
| tr\|Q2FY21\|Q2FY21_STAA8 | 30.5 | 19 | 35 | Penicillin-binding protein 3 OS=Staphylococcus aureus (strain NCTC 8325) GN=SAOUHSC_01652 PE=4 SV=1 | | | 29.0971 | 28.6198 |
| tr\|Q2FXK2\|Q2FXK2_STAA8 | 39.6 | 16 | 35 | Uncharacterized protein OS=Staphylococcus aureus (strain NCTC 8325) GN=SAOUHSC_01832 PE=3 SV=1 | | 23.065 | 29.5029 | 29.5547 |
| sp\|Q2FW10\|RS19_STAA8 | 48.9 | 5 | 34 | 30S ribosomal protein S19 OS=Staphylococcus aureus (strain NCTC 8325) GN=rpsS PE=1 SV=1 | 28.9136 | 29.2278 | 24.7323 | 26.0645 |
| tr\|Q2FZ54\|Q2FZ54_STAA8 | 34.1 | 13 | 34 | Malonyl CoA-acyl carrier protein transacylase OS=Staphylococcus aureus (strain NCTC 8325) G | 28.461 | 28.2222 | 26.7034 | 26.7048 |
| sp\|Q2FXJ5\|SYY_STAA8 | 27.1 | 10 | 34 | Tyrosine--tRNA ligase OS=Staphylococcus aureus (strain NCTC 8325) GN=tyrS PE=3 SV=1 | 27.9475 | 28.9553 | 27.3903 | 26.9462 |
| sp\|Q2FUQ3\|MNMG_STAA8 | 28.6 | 15 | 34 | tRNA uridine 5-carboxymethylaminomethyl modification enzyme MnmG OS=Staphylococcus a | 27.4055 | 27.8153 | 26.7397 | 26.1718 |
| tr\|Q2FV76\|Q2FV76_STAA8 | 36.6 | 17 | 34 | HMG-CoA synthase, putative OS=Staphylococcus aureus (strain NCTC 8325) GN=SAOUHSC_02 | 25.2012 | 25.7692 | 28.4781 | 28.0881 |
| sp\|Q2FWD0\|RPOE_STAA8 | 45.5 | 14 | 33 | Probable DNA-directed RNA polymerase subunit delta OS=Staphylococcus aureus (strain NCTC | 28.4205 | 28.6164 | | |
| tr\|Q2FZR9\|Q2FZR9_STAA8 | 28.3 | 12 | 33 | 3-oxoacyl-[acyl-carrier-protein] synthase 2 OS=Staphylococcus aureus (strain NCTC 8325) GN= | 26.8199 | 26.9539 | 27.581 | 27.8091 |
| tr\|Q2FXQ3\|Q2FXQ3_STAA8 | 55.4 | 9 | 33 | Uncharacterized protein OS=Staphylococcus aureus (strain NCTC 8325) GN=SAOUHSC_01782 | 24.8568 | 24.937 | 30.4884 | 30.5533 |
| tr\|Q2FW50\|Q2FW50_STAA8 | 48.7 | 14 | 32 | Alpha-acetolactate decarboxylase OS=Staphylococcus aureus (strain NCTC 8325) GN=SAOUHS | 27.9019 | 27.6062 | 28.1337 | 27.0872 |
| sp\|Q2FXC7\|SPLE_STAA8 | 37.8 | 12 | 32 | Serine protease SplE OS=Staphylococcus aureus (strain NCTC 8325) GN=splE PE=2 SV=1 | | | 29.7169 | 29.7424 |
| tr\|Q2FX30\|Q2FX30_STAA8 | 78.0 | 9 | 31 | Conserved hypothetical phage protein OS=Staphylococcus aureus (strain NCTC 8325) GN=SAC | 29.6165 | 28.8401 | | |
| tr\|Q2FX98\|Q2FX98_STAA8 | 52.6 | 10 | 31 | Uncharacterized protein OS=Staphylococcus aureus (strain NCTC 8325) GN=SAOUHSC_01979 | 28.0909 | 28.3518 | 25.7886 | 25.7837 |
| tr\|Q2FXI0\|Q2FXI0_STAA8 | 58.2 | 13 | 30 | D-alanine aminotransferase OS=Staphylococcus aureus (strain NCTC 8325) GN=SAOUHSC_018 | 27.6215 | 27.6597 | 24.2397 | 25.0096 |
| tr\|Q2FZU6\|Q2FZU6_STAA8 | 40.9 | 13 | 29 | Multifunctional fusion protein OS=Staphylococcus aureus (strain NCTC 8325) GN=rocD PE=3 S | 27.172 | 27.6098 | 25.0144 | 24.0683 |
| sp\|P52078\|Y997_STAA8 | 40.0 | 12 | 29 | Uncharacterized protein SAOUHSC_00997 OS=Staphylococcus aureus (strain NCTC 8325) GN= | 25.6849 | 25.5639 | 28.8333 | 28.7464 |
| sp\|O05204\|AHPF_STAA8 | 21.3 | 10 | 28 | Alkyl hydroperoxide reductase subunit F OS=Staphylococcus aureus (strain NCTC 8325) GN=ah | 27.1639 | 26.758 | 27.811 | 26.892 |
| tr\|Q2G1M1\|Q2G1M1_STAA8 | 41.5 | 11 | 28 | 3-ketoacyl-acyl carrier protein reductase, putative OS=Staphylococcus aureus (strain NCTC 83 | 27.0543 | 26.8026 | 28.6957 | 28.0846 |
| tr\|Q2FYS0\|Q2FYS0_STAA8 | 29.7 | 11 | 28 | Uncharacterized protein OS=Staphylococcus aureus (strain NCTC 8325) GN=SAOUHSC_01365 | 25.5987 | 24.8921 | 27.8941 | 27.8941 |
| tr\|Q2FV27\|Q2FV27_STAA8 | 57.0 | 11 | 27 | Uncharacterized protein OS=Staphylococcus aureus (strain NCTC 8325) GN=SAOUHSC_02912 | 29.7666 | 29.3468 | | |
| contam_sp\|Q99456\|K1CL_HUMAN | 6.5 | 5 | 27 | Keratin, type I cytoskeletal 12 (Cytokeratin 12) (K12) (CK 12) - Homo sapiens (Human). MW: 5 | 29.6023 | 30.3491 | 29.2815 | 29.1124 |
| sp\|Q2FXQ1\|RL20_STAA8 | 23.7 | 7 | 27 | 50S ribosomal protein L20 OS=Staphylococcus aureus (strain NCTC 8325) GN=rplT PE=1 SV=1 | 28.6685 | 28.4296 | 26.0555 | 26.3585 |
| tr\|Q2FYM3\|Q2FYM3_STAA8 | 42.8 | 12 | 27 | Uncharacterized protein OS=Staphylococcus aureus (strain NCTC 8325) GN=SAOUHSC_01415 | 28.3127 | 28.2557 | 26.3919 | 26.0547 |
| sp\|Q2G1J0\|ALDA_STAA8 | 25.1 | 13 | 27 | Putative aldehyde dehydrogenase AldA OS=Staphylococcus aureus (strain NCTC 8325) GN=ald | 27.5007 | 27.7365 | 26.3561 | 25.105 |
| sp\|Q2G1C0\|TARI1_STAA8 | 40.8 | 10 | 27 | Ribitol-5-phosphate cytidylyltransferase 1 OS=Staphylococcus aureus (strain NCTC 8325) GN= | 26.0167 | 26.4441 | 26.1832 | 28.2343 |
| sp\|P14738\|FNBA_STAA8 | 11.4 | 8 | 26 | Fibronectin-binding protein A OS=Staphylococcus aureus (strain NCTC 8325) GN=fnbA PE=1 S\ | 29.1069 | 29.3724 | | |
| sp\|Q2FXS8\|RL21_STAA8 | 66.7 | 8 | 26 | 50S ribosomal protein L21 OS=Staphylococcus aureus (strain NCTC 8325) GN=rplU PE=1 SV=1 | 28.7001 | 28.9804 | 23.8584 | |
| tr\|Q2G1S3\|Q2G1S3_STAA8 | 32.6 | 12 | 26 | Adenylosuccinate synthetase OS=Staphylococcus aureus (strain NCTC 8325) GN=purA PE=3 SV | 27.3117 | 26.3976 | 25.6247 | 26.4493 |
| tr\|Q2G105\|Q2G105_STAA8 | 47.9 | 12 | 24 | Uncharacterized protein OS=Staphylococcus aureus (strain NCTC 8325) GN=SAOUHSC_00356 | 28.2184 | 28.0685 | | |
| sp\|Q2FZZ8\|GCSH_STAA8 | 37.3 | 5 | 24 | Glycine cleavage system H protein OS=Staphylococcus aureus (strain NCTC 8325) GN=gcvH PE | 27.9525 | 28.1967 | 25.9 | 26.4265 |
| tr\|Q2FX34\|Q2FX34_STAA8 | 60.8 | 12 | 24 | Single-strand DNA-binding protein, putative OS=Staphylococcus aureus (strain NCTC 8325) GN | 27.6698 | 27.9536 | | |
| sp\|Q2FXL7\|DHA2_STAA8 | 35.5 | 13 | 24 | Alanine dehydrogenase 2 OS=Staphylococcus aureus (strain NCTC 8325) GN=ald2 PE=3 SV=1 | 27.4055 | 27.5954 | 25.0746 | 24.1352 |
| tr\|Q2FWV3\|Q2FWV3_STAA8 | 52.1 | 10 | 24 | Staphylokinase, putative OS=Staphylococcus aureus (strain NCTC 8325) GN=SAOUHSC_02171 | 25.2341 | 26.5306 | 28.6759 | 28.071 |
| sp\|Q2FW23\|RS5_STAA8 | 50.0 | 9 | 23 | 30S ribosomal protein S5 OS=Staphylococcus aureus (strain NCTC 8325) GN=rpsE PE=1 SV=1 | 29.0576 | 28.8108 | 27.1918 | 27.2131 |
| sp\|Q2FWN3\|CH10_STAA8 | 57.4 | 6 | 23 | 10 kDa chaperonin OS=Staphylococcus aureus (strain NCTC 8325) GN=groS PE=3 SV=1 | 28.5967 | 28.6657 | 26.5673 | 26.4926 |
| sp\|Q2G081\|QUEF_STAA8 | 49.4 | 8 | 23 | NADPH-dependent 7-cyano-7-deazaguanine reductase OS=Staphylococcus aureus (strain NCT | 27.5269 | 27.6425 | 25.5813 | 24.8103 |
| tr\|Q2G0D4\|Q2G0D4_STAA8 | 19.6 | 5 | 23 | Secretory antigen SsaA-like protein OS=Staphylococcus aureus (strain NCTC 8325) GN=SAOUH | 27.0279 | 26.8734 | 27.3794 | 29.0788 |
| sp\|P0A0F4\|RL11_STAA8 | 42.1 | 8 | 23 | 50S ribosomal protein L11 OS=Staphylococcus aureus (strain NCTC 8325) GN=rplK PE=3 SV=2 | 27.0279 | 27.6801 | 25.4564 | 25.9539 |
| sp\|Q2FW20\|RS8_STAA8 | 50.8 | 7 | 23 | 30S ribosomal protein S8 OS=Staphylococcus aureus (strain NCTC 8325) GN=rpsH PE=1 SV=1 | 26.9553 | 26.8697 | 27.1791 | 27.1071 |
| sp\|Q2G064\|PEPT_STAA8 | 27.9 | 9 | 23 | Peptidase T OS=Staphylococcus aureus (strain NCTC 8325) GN=pepT PE=3 SV=1 | 26.83 | 27.3317 | 27.1791 | 27.2991 |
| sp\|P0A0F8\|RL15_STAA8 | 49.3 | 9 | 22 | 50S ribosomal protein L15 OS=Staphylococcus aureus (strain NCTC 8325) GN=rplO PE=1 SV=1 | 28.9885 | 29.18 | 24.3008 | 24.9238 |
| tr\|Q2FVX4\|Q2FVX4_STAA8 | 34.2 | 14 | 22 | Molybdenum ABC transporter, periplasmic molybdate-binding protein OS=Staphylococcus au | 28.3364 | 27.8222 | | |
| tr\|Q2G071\|Q2G071_STAA8 | 34.5 | 12 | 22 | Uncharacterized protein OS=Staphylococcus aureus (strain NCTC 8325) GN=SAOUHSC_00749 | 27.7845 | 27.4218 | | |
| tr\|Q2FZ04\|Q2FZ04_STAA8 | 37.8 | 12 | 22 | Uncharacterized protein OS=Staphylococcus aureus (strain NCTC 8325) GN=SAOUHSC_01267 | 27.4194 | 28.6279 | 24.336 | 25.1639 |
| sp\|Q2G0S5\|SP5G_STAA8 | 48.0 | 5 | 22 | Putative septation protein SpoVG OS=Staphylococcus aureus (strain NCTC 8325) GN=spoVG P | 27.4194 | 27.8512 | | 24.018 |
| sp\|Q2FYM1\|ODO1_STAA8 | 16.8 | 12 | 22 | 2-oxoglutarate dehydrogenase E1 component OS=Staphylococcus aureus (strain NCTC 8325) ( | 26.6384 | 27.3154 | 26.4804 | 25.6516 |
| tr\|Q2FXM5\|Q2FXM5_STAA8 | 36.7 | 11 | 22 | NADP-dependent malic enzyme, putative OS=Staphylococcus aureus (strain NCTC 8325) GN=S | 26.627 | 27.1841 | | |
| tr\|Q2FYP2\|Q2FYP2_STAA8 | 23.1 | 10 | 22 | ABC transporter, ATP-binding protein, putative OS=Staphylococcus aureus (strain NCTC 8325) | 26.5453 | 25.8161 | 26.2841 | 24.4849 |
| sp\|Q2FZP9\|Y951_STAA8 | 44.4 | 7 | 22 | Putative phosphoesterase SAOUHSC_00951 OS=Staphylococcus aureus (strain NCTC 8325) GN | 26.0755 | 26.2784 | 26.4538 | 26.275 |
| tr\|Q2FZJ7\|Q2FZJ7_STAA8 | 42.9 | 8 | 22 | Uncharacterized protein OS=Staphylococcus aureus (strain NCTC 8325) GN=SAOUHSC_01005 | 23.134 | 25.3194 | 28.4499 | 27.9555 |
| tr\|Q2G2M0\|Q2G2M0_STAA8 | 82.0 | 6 | 21 | Tautomerase OS=Staphylococcus aureus (strain NCTC 8325) GN=SAOUHSC_01362 PE=3 SV=1 | 30.3343 | 29.7314 | 27.9722 | 27.8331 |
| sp\|P48860\|RL7_STAA8 | 44.3 | 4 | 21 | 50S ribosomal protein L7/L12 OS=Staphylococcus aureus (strain NCTC 8325) GN=rplL PE=3 SV | 27.8389 | 28.3015 | 26.1209 | 25.2963 |
| tr\|Q2FZZ0\|Q2FZZ0_STAA8 | 38.5 | 10 | 21 | Lipoprotein OS=Staphylococcus aureus (strain NCTC 8325) GN=SAOUHSC_00844 PE=3 SV=1 | 26.3276 | 26.0349 | 26.6997 | 27.3183 |
| tr\|Q2FX05\|Q2FX05_STAA8 | 30.2 | 7 | 21 | Methionine aminopeptidase OS=Staphylococcus aureus (strain NCTC 8325) GN=map PE=3 SV= | 25.7803 | 26.0947 | 28.2218 | 28.6611 |
| tr\|Q2FY68\|Q2FY68_STAA8 | 37.9 | 9 | 21 | Pyrroline-5-carboxylate reductase OS=Staphylococcus aureus (strain NCTC 8325) GN=proC PE= | 25.1013 | 25.7147 | 26.2186 | 26.4671 |
| sp\|Q2FZI8\|PUR5_STAA8 | 30.7 | 10 | 21 | Phosphoribosylformylglycinamidine cyclo-ligase OS=Staphylococcus aureus (strain NCTC 8325 | 25.0504 | 26.7334 | 25.5041 | 26.6902 |
| sp\|Q2FYK5\|TYSY_STAA8 | 35.2 | 13 | 20 | Thymidylate synthase OS=Staphylococcus aureus (strain NCTC 8325) GN=thyA PE=3 SV=1 | 27.1309 | 27.1157 | 24.5688 | |
| tr\|Q2G0L8\|Q2G0L8_STAA8 | 28.0 | 7 | 19 | Uncharacterized protein OS=Staphylococcus aureus (strain NCTC 8325) GN=SAOUHSC_00542 | 28.8408 | 27.6944 | 24.2767 | 24.283 |
| sp\|Q9RFJ6\|ROT_STAA8 | 33.1 | 8 | 19 | HTH-type transcriptional regulator rot OS=Staphylococcus aureus (strain NCTC 8325) GN=rot I | 28.2967 | 28.4309 | | |
| tr\|Q2G0S0\|Q2G0S0_STAA8 | 32.7 | 8 | 19 | 50S ribosomal protein L25 OS=Staphylococcus aureus (strain NCTC 8325) GN=rplY PE=1 SV=1 | 27.907 | 28.3556 | 26.9433 | 25.5495 |
| sp\|Q2G0L1\|GCH4_STAA8 | 35.3 | 11 | 19 | GTP cyclohydrolase FolE2 OS=Staphylococcus aureus (strain NCTC 8325) GN=folE2 PE=3 SV=1 | 27.5591 | 27.5712 | 25.3342 | 25.6415 |
| tr\|Q2FZZ9\|Q2FZZ9_STAA8 | 76.3 | 8 | 19 | Uncharacterized protein OS=Staphylococcus aureus (strain NCTC 8325) GN=SAOUHSC_00835 | 27.3914 | 27.4263 | 27.0017 | 25.8389 |
| sp\|Q2FVR9\|IDI2_STAA8 | 21.5 | 9 | 19 | Isopentenyl-diphosphate delta-isomerase OS=Staphylococcus aureus (strain NCTC 8325) GN=I | 27.2433 | 28.3743 | 24.2397 | 24.8291 |
| tr\|Q2G0Z9\|Q2G0Z9_STAA8 | 36.5 | 9 | 19 | Uncharacterized protein OS=Staphylococcus aureus (strain NCTC 8325) GN=SAOUHSC_00362 | 27.0011 | 26.8364 | | |
| tr\|Q2FYB2\|Q2FYB2_STAA8 | 49.4 | 10 | 18 | Bacteriophage L54a, deoxyuridine 5-triphosphate nucleotidohydrolase OS=Staphylococcus au | 29.3685 | 29.2408 | | |
| sp\|Q2FW39\|RS9_STAA8 | 49.2 | 7 | 18 | 30S ribosomal protein S9 OS=Staphylococcus aureus (strain NCTC 8325) GN=rpsI PE=1 SV=1 | 27.7678 | 28.3114 | 26.6057 | 26.9639 |
| sp\|Q2FY16\|END4_STAA8 | 23.0 | 7 | 18 | Probable endonuclease 4 OS=Staphylococcus aureus (strain NCTC 8325) GN=nfo PE=3 SV=1 | 27.3843 | 27.7722 | 26.7217 | 25.7735 |
| sp\|Q2G2M6\|SYC_STAA8 | 20.4 | 9 | 18 | Cysteine--tRNA ligase OS=Staphylococcus aureus (strain NCTC 8325) GN=cysS PE=3 SV=1 | 27.3556 | 26.7656 | 23.4824 | 23.7472 |
| sp\|Q2FZX0\|NAGD_STAA8 | 19.3 | 7 | 18 | Acid sugar phosphatase OS=Staphylococcus aureus (strain NCTC 8325) GN=nagD PE=3 SV=1 | 27.2967 | 27.5586 | 26.2399 | 27.1713 |
| tr\|Q2FZJ5\|Q2FZJ5_STAA8 | 55.4 | 8 | 18 | N5-carboxyaminoimidazole ribonucleotide mutase OS=Staphylococcus aureus (strain NCTC 83 | 26.2717 | 27.3705 | 26.5256 | 26.4498 |
| sp\|Q2G1W2\|PCKA_STAA8 | 23.2 | 10 | 18 | Phosphoenolpyruvate carboxykinase (ATP) OS=Staphylococcus aureus (strain NCTC 8325) GN= | 25.3617 | 26.6369 | 26.4012 | 24.3287 |
| sp\|Q2FXC4\|SPLC_STAA8 | 26.8 | 6 | 18 | Serine protease SplC OS=Staphylococcus aureus (strain NCTC 8325) GN=splC PE=1 SV=1 | | | 29.279 | 29.0189 |
| sp\|Q2FWK3\|LEU1_STAA8 | 21.6 | 11 | 18 | 2-isopropylmalate synthase OS=Staphylococcus aureus (strain NCTC 8325) GN=leuA PE=3 SV=1 | | | 27.7552 | 27.1866 |
| sp\|Q2G045\|HPRK_STAA8 | 38.7 | 10 | 17 | HPr kinase/phosphorylase OS=Staphylococcus aureus (strain NCTC 8325) GN=hprK PE=3 SV=1 | 29.4743 | 27.9664 | 26.1942 | 23.5526 |
| tr\|Q2FY27\|Q2FY27_STAA8 | 26.5 | 9 | 17 | Glucokinase, putative OS=Staphylococcus aureus (strain NCTC 8325) GN=SAOUHSC_01646 PE | 28.4353 | 28.9556 | | |
| tr\|Q2G1T5\|Q2G1T5_STAA8 | 10.8 | 8 | 17 | Fibronectin binding protein B, putative OS=Staphylococcus aureus (strain NCTC 8325) GN=SAC | 28.231 | 28.4648 | | |
| tr\|Q2FXL3\|Q2FXL3_STAA8 | 40.9 | 6 | 17 | Probable thiol peroxidase OS=Staphylococcus aureus (strain NCTC 8325) GN=tpx PE=1 SV=1 | 27.4264 | 27.7961 | 26.2738 | 26.7694 |
| sp\|P47768\|RPOB_STAA8 | 13.4 | 12 | 17 | DNA-directed RNA polymerase subunit beta OS=Staphylococcus aureus (strain NCTC 8325) GN | 26.9829 | 25.8708 | 24.7659 | 23.3201 |
| sp\|Q2FZ42\|RL19_STAA8 | 28.4 | 4 | 17 | 50S ribosomal protein L19 OS=Staphylococcus aureus (strain NCTC 8325) GN=rplS PE=1 SV=1 | 26.8499 | 25.1243 | 26.9217 | 27.1659 |
| tr\|Q2G235\|Q2G235_STAA8 | 16.5 | 8 | 17 | Nicotinate phosphoribosyltransferase OS=Staphylococcus aureus (strain NCTC 8325) GN=SAO | 25.0746 | 26.0404 | 25.8256 | 26.8918 |
| sp\|O34090\|HEM3_STAA8 | 27.3 | 7 | 16 | Porphobilinogen deaminase OS=Staphylococcus aureus (strain NCTC 8325) GN=hemC PE=3 SV | 28.7001 | 28.2374 | 24.8068 | 25.071 |
| sp\|Q2FXL9\|Y1816_STAA8 | 19.4 | 6 | 16 | Uncharacterized peptidase SAOUHSC_01816 OS=Staphylococcus aureus (strain NCTC 8325) G | 27.5654 | 27.839 | 25.3606 | |
| sp\|Q2G015\|CLFA_STAA8 | 9.3 | 8 | 16 | Clumping factor A OS=Staphylococcus aureus (strain NCTC 8325) GN=clfA PE=1 SV=1 | 26.8598 | 26.3783 | 23.8916 | |
| tr\|Q2G2D8\|Q2G2D8_STAA8 | 36.5 | 11 | 16 | ABC transporter, substrate-binding protein, putative OS=Staphylococcus aureus (strain NCTC 8 | 26.83 | 27.1047 | | 23.9701 |
| sp\|Q2G0Y7\|IMDH_STAA8 | 26.6 | 10 | 16 | Inosine-5'-monophosphate dehydrogenase OS=Staphylococcus aureus (strain NCTC 8325) GN | 26.8199 | 25.563 | 24.4364 | 23.6924 |
| tr\|Q2FW75\|Q2FW75_STAA8 | 23.5 | 7 | 16 | ABC transporter periplasmic binding protein, putative OS=Staphylococcus aureus (strain NCTC | 26.4718 | 25.6696 | | 25.6824 |
| sp\|Q2FWG0\|TENA_STAA8 | 16.6 | 4 | 16 | Aminopyrimidine aminohydrolase OS=Staphylococcus aureus (strain NCTC 8325) GN=tenA PE | 26.3815 | 26.7698 | 28.2718 | 27.9303 |

| | | | | | | | | |
|---|---|---|---|---|---|---|---|---|
| sp\|Q2FW33\|RL17_STAA8 | 32.8 | 5 | 16 | 50S ribosomal protein L17 OS=Staphylococcus aureus (strain NCTC 8325) GN=rplQ PE=1 SV=1 | 26.0755 | 25.9875 | 28.4162 | 27.2814 |
| tr\|Q2G2D2\|Q2G2D2_STAA8 | 18.2 | 5 | 16 | Transcription termination/antitermination protein NusA OS=Staphylococcus aureus (strain NC | 25.7995 | 26.5521 | | |
| tr\|Q2FVX8\|Q2FVX8_STAA8 | 42.3 | 7 | 16 | Molybdenum cofactor biosynthesis protein B OS=Staphylococcus aureus (strain NCTC 8325) G | 25.7899 | 25.3771 | 27.0162 | 27.0013 |
| tr\|Q2FZG6\|Q2FZG6_STAA8 | 43.7 | 7 | 16 | Peptide deformylase OS=Staphylococcus aureus (strain NCTC 8325) GN=def PE=3 SV=1 | 25.577 | 25.1427 | 26.8044 | 26.6953 |
| tr\|Q2FWW3\|Q2FWW3_STAA8 | 17.3 | 7 | 16 | Uncharacterized protein OS=Staphylococcus aureus (strain NCTC 8325) GN=SAOUHSC_02158 | 24.5351 | 25.6603 | 27.335 | 27.2697 |
| sp\|Q2FWG2\|THIM_STAA8 | 21.3 | 5 | 15 | Hydroxyethylthiazole kinase OS=Staphylococcus aureus (strain NCTC 8325) GN=thiM PE=3 SV= | 28.7218 | 28.5958 | | |
| sp\|Q2G0N9\|RL10_STAA8 | 54.2 | 7 | 15 | 50S ribosomal protein L10 OS=Staphylococcus aureus (strain NCTC 8325) GN=rplJ PE=3 SV=1 | 27.8174 | 28.213 | | |
| sp\|Q2FVA4\|Y2829_STAA8 | 46.2 | 8 | 15 | Putative NAD(P)H nitroreductase SAOUHSC_02829 OS=Staphylococcus aureus (strain NCTC 83 | 27.0716 | 26.9553 | | |
| sp\|Q2G2Q0\|GYRA_STAA8 | 12.1 | 11 | 15 | DNA gyrase subunit A OS=Staphylococcus aureus (strain NCTC 8325) GN=gyrA PE=1 SV=1 | 26.5212 | 26.612 | | |
| sp\|Q2FZG9\|RNJ1_STAA8 | 18.6 | 11 | 15 | Ribonuclease J 1 OS=Staphylococcus aureus (strain NCTC 8325) GN=rnj1 PE=1 SV=1 | 26.0755 | 26.262 | | |
| sp\|Q2G0N5\|RPOC_STAA8 | 9.6 | 11 | 15 | DNA-directed RNA polymerase subunit beta' OS=Staphylococcus aureus (strain NCTC 8325) GI | 25.7668 | 25.7726 | 25.4119 | 26.5929 |
| tr\|Q2FZ15\|Q2FZ15_STAA8 | 22.4 | 10 | 15 | Uncharacterized protein OS=Staphylococcus aureus (strain NCTC 8325) GN=SAOUHSC_01256 | 25.0686 | 25.974 | 26.2452 | 23.9481 |
| sp\|Q2FZ25\|RS2_STAA8 | 31.0 | 7 | 14 | 30S ribosomal protein S2 OS=Staphylococcus aureus (strain NCTC 8325) GN=rpsB PE=1 SV=2 | 28.1799 | 27.2756 | | |
| tr\|Q2FZ36\|Q2FZ36_STAA8 | 29.1 | 5 | 14 | Succinate--CoA ligase [ADP-forming] subunit alpha OS=Staphylococcus aureus (strain NCTC 83 | 27.6277 | 27.054 | | 24.1521 |
| tr\|Q2G1Z0\|Q2G1Z0_STAA8 | 20.5 | 5 | 14 | Uncharacterized protein OS=Staphylococcus aureus (strain NCTC 8325) GN=SAOUHSC_00655 | 26.9553 | 27.8326 | 25.3865 | 26.5756 |
| tr\|Q2G2T1\|Q2G2T1_STAA8 | 19.5 | 7 | 14 | Uncharacterized protein OS=Staphylococcus aureus (strain NCTC 8325) GN=SAOUHSC_01973 | 26.8987 | 25.6138 | | |
| sp\|Q2FW12\|RS3_STAA8 | 32.3 | 7 | 14 | 30S ribosomal protein S3 OS=Staphylococcus aureus (strain NCTC 8325) GN=rpsC PE=1 SV=1 | 26.3682 | 26.7557 | | |
| tr\|Q2FYF9\|Q2FYF9_STAA8 | 26.1 | 8 | 14 | 30S ribosomal protein S1, putative OS=Staphylococcus aureus (strain NCTC 8325) GN=SAOUH | 26.1841 | 26.3535 | 26.3391 | 25.1762 |
| tr\|Q2G220\|Q2G220_STAA8 | 33.9 | 6 | 14 | Uncharacterized protein OS=Staphylococcus aureus (strain NCTC 8325) GN=SAOUHSC_02980 | 25.9779 | 25.8965 | 27.1204 | 27.185 |
| tr\|Q2G2U0\|Q2G2U0_STAA8 | 30.8 | 10 | 14 | N-acetylglucosamine-6-phosphate deacetylase OS=Staphylococcus aureus (strain NCTC 8325) | 25.6539 | 24.6859 | 27.4638 | 24.8205 |
| sp\|Q2FW11\|RL22_STAA8 | 45.3 | 5 | 14 | 50S ribosomal protein L22 OS=Staphylococcus aureus (strain NCTC 8325) GN=rplV PE=1 SV=1 | 25.3617 | 23.851 | 24.4682 | 24.7339 |
| tr\|Q2FZ83\|Q2FZ83_STAA8 | 41.0 | 7 | 13 | Uncharacterized protein OS=Staphylococcus aureus (strain NCTC 8325) GN=SAOUHSC_01158 | 29.9098 | 30.183 | 24.4992 | 24.5724 |
| sp\|Q2FW14\|RL29_STAA8 | 50.7 | 4 | 13 | 50S ribosomal protein L29 OS=Staphylococcus aureus (strain NCTC 8325) GN=rpmC PE=1 SV=1 | 27.212 | 27.1079 | 26.2452 | 25.942 |
| tr\|Q2G1N4\|Q2G1N4_STAA8 | 15.8 | 5 | 13 | Periplasmic binding protein, putative OS=Staphylococcus aureus (strain NCTC 8325) GN=SAOU | 26.5333 | 26.8634 | 24.9148 | 25.0854 |
| tr\|Q2G115\|Q2G115_STAA8 | 30.4 | 8 | 13 | Ribosome-binding ATPase YchF OS=Staphylococcus aureus (strain NCTC 8325) GN=ychF PE=3 | 26.3947 | 26.1738 | | |
| tr\|Q2G0M1\|Q2G0M1_STAA8 | 33.2 | 6 | 13 | Uncharacterized protein OS=Staphylococcus aureus (strain NCTC 8325) GN=SAOUHSC_00539 | 25.5682 | 25.2628 | 24.359 | 24.596 |
| sp\|Q2FYU4\|GUAC_STAA8 | 24.9 | 6 | 13 | GMP reductase OS=Staphylococcus aureus (strain NCTC 8325) GN=guaC PE=3 SV=1 | 25.4892 | 26.3867 | | |
| sp\|Q2FWE8\|ATPA_STAA8 | 15.5 | 6 | 13 | ATP synthase subunit alpha OS=Staphylococcus aureus (strain NCTC 8325) GN=atpA PE=3 SV= | 23.5705 | 24.4481 | 27.135 | 31.2332 |
| tr\|Q2FV10\|Q2FV10_STAA8 | 13.7 | 6 | 13 | Betaine aldehyde dehydrogenase OS=Staphylococcus aureus (strain NCTC 8325) GN=SAOUHS | 21.2015 | | 27.4117 | 27.2357 |
| tr\|Q2G107\|Q2G107_STAA8 | 27.6 | 5 | 13 | Uncharacterized protein OS=Staphylococcus aureus (strain NCTC 8325) GN=SAOUHSC_00354 PE=4 SV=1 | | | 27.4638 | 28.2209 |
| sp\|Q2FXC2\|SPLA_STAA8 | 19.1 | 4 | 13 | Serine protease SplA OS=Staphylococcus aureus (strain NCTC 8325) GN=splA PE=1 SV=1 | | | 28.1398 | 28.2842 |
| sp\|Q2G0D0\|Y675_STAA8 | 27.3 | 6 | 12 | Probable transcriptional regulatory protein SAOUHSC_00675 OS=Staphylococcus aureus (strai | 28.2642 | 27.0065 | 27.0032 | 26.9605 |
| sp\|Q2FZ19\|RNJ2_STAA8 | 15.1 | 6 | 12 | Ribonuclease J 2 OS=Staphylococcus aureus (strain NCTC 8325) GN=rnj2 PE=1 SV=2 | 26.0497 | 25.4893 | 24.8307 | |
| tr\|Q2G0Q6\|Q2G0Q6_STAA8 | 39.7 | 3 | 12 | 7,8-dihydroneopterin aldolase OS=Staphylococcus aureus (strain NCTC 8325) GN=SAOUHSC_( | 26.005 | 26.3977 | | 24.0861 |
| sp\|Q2FY41\|EFP_STAA8 | 29.2 | 4 | 12 | Elongation factor P OS=Staphylococcus aureus (strain NCTC 8325) GN=efp PE=3 SV=1 | 25.5857 | 25.9811 | 24.8773 | |
| tr\|Q2FXY2\|Q2FXY2_STAA8 | 49.0 | 5 | 12 | Uncharacterized protein OS=Staphylococcus aureus (strain NCTC 8325) GN=SAOUHSC_01698 | 24.9565 | 26.4822 | 25.1932 | 24.5022 |
| tr\|Q2FZS2\|Q2FZS2_STAA8 | 36.1 | 5 | 12 | Truncated MHC class II analog protein OS=Staphylococcus aureus (strain NCTC 8325) GN=SAO | 24.5393 | 24.6154 | 26.9631 | 26.9238 |
| tr\|Q2G123\|Q2G123_STAA8 | 14.0 | 4 | 12 | Uncharacterized protein OS=Staphylococcus aureus (strain NCTC 8325) GN=SAOUHSC_00337 | 23.8568 | 23.8633 | 27.335 | 27.5582 |
| tr\|Q2FVK3\|Q2FVK3_STAA8 | 17.5 | 6 | 12 | Gamma-hemolysin h-gamma-ii subunit, putative OS=Staphylococcus aureus (strain NCTC 8325) GN=SAOUHSC_027 | | | 29.117 | 28.7751 |
| tr\|Q2FZ03\|Q2FZ03_STAA8 | 46.4 | 6 | 11 | Uncharacterized protein OS=Staphylococcus aureus (strain NCTC 8325) GN=SAOUHSC_01268 | 27.1309 | 27.3043 | | |
| tr\|Q2FZ62\|Q2FZ62_STAA8 | 17.8 | 5 | 11 | Ribulose-phosphate 3-epimerase OS=Staphylococcus aureus (strain NCTC 8325) GN=SAOUHS( | 26.8793 | 25.6439 | 27.0061 | 27.3161 |
| tr\|Q2FYI6\|Q2FYI6_STAA8 | 16.8 | 5 | 11 | Uncharacterized protein OS=Staphylococcus aureus (strain NCTC 8325) GN=SAOUHSC_01460 | 26.5808 | 26.2887 | | |
| tr\|Q2FZL5\|Q2FZL5_STAA8 | 21.6 | 5 | 11 | 1,4-dihydroxy-2-naphthoyl-CoA synthase OS=Staphylococcus aureus (strain NCTC 8325) GN=m | 26.2859 | 26.2827 | 25.2227 | 24.7877 |
| sp\|Q2FYP3\|CVFB_STAA8 | 26.3 | 6 | 11 | Conserved virulence factor B OS=Staphylococcus aureus (strain NCTC 8325) GN=cvfB PE=1 SV= | 26.0233 | 26.7439 | | |
| sp\|O07325\|FTSA_STAA8 | 18.1 | 7 | 11 | Cell division protein FtsA OS=Staphylococcus aureus (strain NCTC 8325) GN=ftsA PE=1 SV=2 | 25.5505 | 26.0155 | | |
| sp\|Q2FW66\|ASP23_STAA8 | 20.7 | 5 | 10 | Alkaline shock protein 23 OS=Staphylococcus aureus (strain NCTC 8325) GN=asp23 PE=3 SV=1 | 27.5654 | 28.3167 | | |
| tr\|Q2G0I4\|Q2G0I4_STAA8 | 22.0 | 8 | 10 | Uncharacterized protein OS=Staphylococcus aureus (strain NCTC 8325) GN=SAOUHSC_00581 | 27.2041 | 24.5552 | 25.8545 | 25.8158 |
| tr\|Q2G0G6\|Q2G0G6_STAA8 | 36.2 | 7 | 10 | Uncharacterized protein OS=Staphylococcus aureus (strain NCTC 8325) GN=SAOUHSC_00603 | 26.6156 | 26.5364 | 23.0693 | |
| tr\|Q2FZC2\|Q2FZC2_STAA8 | 29.4 | 5 | 10 | Fibrinogen-binding protein-related OS=Staphylococcus aureus (strain NCTC 8325) GN=SAOUH | 26.5212 | 27.6411 | | |
| tr\|Q2FXN9\|Q2FXN9_STAA8 | 10.3 | 6 | 10 | DNA polymerase I OS=Staphylococcus aureus (strain NCTC 8325) GN=polA PE=3 SV=1 | 26.3815 | 26.857 | | |
| tr\|Q2FX90\|Q2FX90_STAA8 | 38.2 | 6 | 10 | Uncharacterized protein OS=Staphylococcus aureus (strain NCTC 8325) GN=SAOUHSC_01987 | 26.2139 | 25.7122 | | |
| sp\|Q2FV39\|Y2900_STAA8 | 28.3 | 6 | 10 | Uncharacterized hydrolase SAOUHSC_02900 OS=Staphylococcus aureus (strain NCTC 8325) GI | 26.1691 | 25.3119 | | 24.0208 |
| sp\|Q2FZ58\|Y1193_STAA8 | 13.3 | 4 | 10 | Uncharacterized protein SAOUHSC_01193 OS=Staphylococcus aureus (strain NCTC 8325) GN= | 25.0776 | 25.6023 | | |
| sp\|Q2G2C4\|TARI2_STAA8 | 15.5 | 4 | 10 | Ribitol-5-phosphate cytidylyltransferase 2 OS=Staphylococcus aureus (strain NCTC 8325) GN= | 24.6405 | 24.7243 | 25.944 | 26.1816 |
| tr\|Q2FXE2\|Q2FXE2_STAA8 | 20.6 | 6 | 10 | Uncharacterized protein OS=Staphylococcus aureus (strain NCTC 8325) GN=SAOUHSC_01907 PE=4 SV=1 | | | 27.7222 | 27.3932 |
| tr\|Q2FYV3\|Q2FYV3_STAA8 | 19.8 | 5 | 10 | Threonine synthase OS=Staphylococcus aureus (strain NCTC 8325) GN=SAOUHSC_01321 PE=3 SV=1 | | | 27.581 | 26.5656 |
| tr\|Q2G112\|Q2G112_STAA8 | 25.7 | 5 | 9 | Single-stranded DNA-binding protein OS=Staphylococcus aureus (strain NCTC 8325) GN=SAOU | 27.2433 | 26.4709 | | 25.1093 |
| sp\|Q2FY33\|GCST_STAA8 | 19.0 | 7 | 9 | Aminomethyltransferase OS=Staphylococcus aureus (strain NCTC 8325) GN=gcvT PE=3 SV=1 | 25.8316 | 25.4516 | | |
| tr\|Q2FVW9\|Q2FVW9_STAA8 | 26.2 | 5 | 9 | Uncharacterized protein OS=Staphylococcus aureus (strain NCTC 8325) GN=SAOUHSC_02554 | 25.8278 | 25.3334 | | |
| sp\|Q2FWE0\|RF1_STAA8 | 16.5 | 4 | 9 | Peptide chain release factor 1 OS=Staphylococcus aureus (strain NCTC 8325) GN=prfA PE=3 SV | 25.2926 | 25.1795 | 23.9208 | |
| sp\|Q2FXW0\|Y1721_STAA8 | 46.5 | 4 | 9 | UPF0297 protein SAOUHSC_01721 OS=Staphylococcus aureus (strain NCTC 8325) GN=SAOUH | 25.2259 | 24.8485 | 27.3574 | 26.8216 |
| sp\|Q2FXM0\|Y1815_STAA8 | 17.0 | 3 | 9 | UPF0173 metal-dependent hydrolase SAOUHSC_01815 OS=Staphylococcus aureus (strain NCT | 24.9436 | 25.8871 | 24.8386 | 24.1675 |
| tr\|Q2FXM3\|Q2FXM3_STAA8 | 24.0 | 5 | 9 | Uncharacterized protein OS=Staphylococcus aureus (strain NCTC 8325) GN=SAOUHSC_01812 | 24.3859 | 26.3289 | 24.6256 | 25.8155 |
| sp\|Q2FYR6\|TRPC_STAA8 | 17.3 | 4 | 9 | Indole-3-glycerol phosphate synthase OS=Staphylococcus aureus (strain NCTC 8325) GN=trpC | 23.0304 | 24.6683 | 26.1382 | 27.391 |
| tr\|Q2FZI9\|Q2FZI9_STAA8 | 19.8 | 7 | 9 | Amidophosphoribosyltransferase OS=Staphylococcus aureus (strain NCTC 8325) GN=purF PE=3 SV=1 | | 23.0772 | 26.3219 | 26.7017 |
| tr\|Q2G1C9\|Q2G1C9_STAA8 | 9.6 | 5 | 9 | Uncharacterized protein OS=Staphylococcus aureus (strain NCTC 8325) GN=SAOUHSC_00196 PE=3 SV=1 | | | 26.2815 | 26.5749 |
| sp\|Q2FWF0\|ATPB_STAA8 | 10.0 | 4 | 8 | ATP synthase subunit beta OS=Staphylococcus aureus (strain NCTC 8325) GN=atpD PE=3 SV=1 | 28.9622 | 28.9769 | 24.3928 | 24.7872 |
| sp\|Q2FZD2\|THIO_STAA8 | 19.2 | 3 | 8 | Thioredoxin OS=Staphylococcus aureus (strain NCTC 8325) GN=trxA PE=2 SV=1 | 28.1971 | 28.7774 | 24.7151 | 28.8954 |
| sp\|Q2FXQ7\|CLPX_STAA8 | 22.9 | 6 | 8 | ATP-dependent Clp protease ATP-binding subunit ClpX OS=Staphylococcus aureus (strain NCT | 25.5122 | 25.2055 | | |
| sp\|Q2FXP9\|IF3_STAA8 | 23.4 | 4 | 8 | Translation initiation factor IF-3 OS=Staphylococcus aureus (strain NCTC 8325) GN=infC PE=3 | 25.2979 | 25.2091 | 26.4425 | 25.9743 |
| tr\|Q2G2T8\|Q2G2T8_STAA8 | 16.5 | 4 | 8 | Uncharacterized protein OS=Staphylococcus aureus (strain NCTC 8325) GN=SAOUHSC_00712 | 25.0443 | 25.2059 | 27.9514 | |
| sp\|Q2FXJ0\|MURC_STAA8 | 15.1 | 5 | 8 | UDP-N-acetylmuramate--L-alanine ligase OS=Staphylococcus aureus (strain NCTC 8325) GN=m | 24.7831 | 25.6933 | | |
| sp\|Q2FVB2\|F16PC_STAA8 | 9.2 | 6 | 8 | Fructose-1,6-bisphosphatase class 3 OS=Staphylococcus aureus (strain NCTC 8325) GN=fbp PE | 22.518 | | 29.8801 | 27.5403 |
| sp\|Q2FYN6\|Y1399_STAA8 | 19.8 | 6 | 8 | Uncharacterized hydrolase SAOUHSC_01399 OS=Staphylococcus aureus (strain NCTC 8325) GN=SAOUHSC_01399 F | | | 25.4516 | 26.8274 |
| tr\|Q2FUW9\|Q2FUW9_STAA8 | 7.6 | 5 | 8 | Uncharacterized protein OS=Staphylococcus aureus (strain NCTC 8325) GN=SAOUHSC_02982 PE=4 SV=1 | | | 25.6625 | 26.4913 |
| tr\|Q2FXH8\|Q2FXH8_STAA8 | 42.1 | 5 | 7 | Uncharacterized protein OS=Staphylococcus aureus (strain NCTC 8325) GN=SAOUHSC_01869 | 27.8864 | 24.4678 | 24 | |
| sp\|Q2FXW2\|Y1719_STAA8 | 13.7 | 3 | 7 | UPF0473 protein SAOUHSC_01719 OS=Staphylococcus aureus (strain NCTC 8325) GN=SAOUH | 25.4518 | 26.2299 | | |
| tr\|Q2FWY2\|Q2FWY2_STAA8 | 21.5 | 5 | 7 | Pyrazinamidase/nicotinamidase, putative OS=Staphylococcus aureus (strain NCTC 8325) GN=! | 25.3642 | 25.7963 | | |
| sp\|Q2FWY8\|GATC_STAA8 | 49.0 | 4 | 7 | Aspartyl/glutamyl-tRNA(Asn/Gln) amidotransferase subunit C OS=Staphylococcus aureus (stra | 25.2821 | 24.8628 | | |
| sp\|Q2FXW7\|GREA_STAA8 | 47.5 | 4 | 7 | Transcription elongation factor GreA OS=Staphylococcus aureus (strain NCTC 8325) GN=greA | 25.1391 | 25.9376 | | |
| sp\|Q2FWJ3\|RSBW_STAA8 | 34.0 | 4 | 7 | Serine-protein kinase RsbW OS=Staphylococcus aureus (strain NCTC 8325) GN=rsbW PE=3 SV= | 25.1042 | 25.1567 | | |
| sp\|Q2G260\|Y094_STAA8 | 18.6 | 3 | 7 | Uncharacterized protein SAOUHSC_00094 OS=Staphylococcus aureus (strain NCTC 8325) GN= | 24.9207 | 23.9536 | 23.645 | |
| sp\|Q2FYG0\|DER_STAA8 | 11.5 | 5 | 7 | GTPase Der OS=Staphylococcus aureus (strain NCTC 8325) GN=der PE=3 SV=1 | 24.8291 | 26.0064 | | |
| sp\|Q2G283\|GSA2_STAA8 | 10.5 | 4 | 7 | Glutamate-1-semialdehyde 2,1-aminomutase 2 OS=Staphylococcus aureus (strain NCTC 8325) | 24.7283 | 25.0122 | | |
| tr\|Q2G2F0\|Q2G2F0_STAA8 | 18.6 | 3 | 7 | Uncharacterized protein OS=Staphylococcus aureus (strain NCTC 8325) GN=SAOUHSC_01968 | 23.5002 | 24.7132 | 24.1558 | |
| sp\|Q2G189\|ESXA_STAA8 | 34.0 | 4 | 7 | ESAT-6 secretion system extracellular protein A OS=Staphylococcus aureus (strain NCTC 8325) GN=esxA PE=1 SV=1 | | | 27.6883 | 27.928 |
| tr\|Q2FUT0\|Q2FUT0_STAA8 | 11.3 | 3 | 7 | Uncharacterized protein OS=Staphylococcus aureus (strain NCTC 8325) GN=SAOUHSC_03021 PE=4 SV=1 | | | 26.7289 | 26.5495 |
| tr\|Q2G0V0\|Q2G0V0_STAA8 | 13.6 | 3 | 6 | Lipoprotein OS=Staphylococcus aureus (strain NCTC 8325) GN=SAOUHSC_00426 PE=3 SV=1 | 27.3984 | 27.7718 | 26.4956 | 25.9207 |
| tr\|Q2FX68\|Q2FX68_STAA8 | 10.8 | 5 | 6 | Phi ETA orf 56-like protein OS=Staphylococcus aureus (strain NCTC 8325) GN=SAOUHSC_0202 | 25.5857 | 25.5369 | | |
| tr\|Q2G0F6\|Q2G0F6_STAA8 | 22.9 | 6 | 6 | Iron compound ABC transporter, substrate-binding protein, putative OS=Staphylococcus aure | 25.4062 | 24.2694 | | |

| | | | | | | | | |
|---|---|---|---|---|---|---|---|---|
| tr\|Q2FUQ9\|Q2FUQ9_STAA8 | 46.7 | 3 | 6 | Cold shock protein, putative OS=Staphylococcus aureus (strain NCTC 8325) GN=SAOUHSC_03 | 25.316 | 24.9936 | | |
| sp\|P72360\|SCDA_STAA8 | 11.6 | 4 | 6 | Iron-sulfur cluster repair protein ScdA OS=Staphylococcus aureus (strain NCTC 8325) GN=scd | 24.7831 | 25.1534 | | |
| tr\|Q2G2D7\|Q2G2D7_STAA8 | 16.2 | 4 | 6 | Uncharacterized protein OS=Staphylococcus aureus (strain NCTC 8325) GN=SAOUHSC_02447 | 24.6208 | 24.8623 | | 24.0724 |
| tr\|Q2FY43\|Q2FY43_STAA8 | 14.9 | 4 | 6 | Acetyl-CoA carboxylase, biotin carboxylase OS=Staphylococcus aureus (strain NCTC 8325) GN= | 24.4753 | 24.8209 | | 26.2497 |
| sp\|Q2G0P2\|NUSG_STAA8 | 23.6 | 3 | 6 | Transcription termination/antitermination protein NusG OS=Staphylococcus aureus (strain N | 24.4177 | 24.3246 | | |
| sp\|Q2FXR4\|GSA1_STAA8 | 14.7 | 5 | 6 | Glutamate-1-semialdehyde 2,1-aminomutase 1 OS=Staphylococcus aureus (strain NCTC 8325 | 24.3996 | 24.0623 | | |
| sp\|P0A0G2\|RL30_STAA8 | 39.0 | 3 | 6 | 50S ribosomal protein L30 OS=Staphylococcus aureus (strain NCTC 8325) GN=rpmD PE=1 SV= | 23.7165 | 25.465 | 24.7742 | |
| tr\|Q2FZ48\|Q2FZ48_STAA8 | 5.0 | 3 | 6 | Signal recognition particle receptor FtsY OS=Staphylococcus aureus (strain NCTC 8325) GN=ft | 23.6083 | 24.7644 | 26.9387 | 25.3525 |
| tr\|Q2FY51\|Q2FY51_STAA8 | 8.9 | 4 | 6 | Dihydrolipoyl dehydrogenase OS=Staphylococcus aureus (strain NCTC 8325) GN=SAOUHSC_0 | 23.0411 | 23.4441 | 26.3044 | |
| tr\|Q2FZN7\|Q2FZN7_STAA8 | 10.4 | 3 | 6 | Lipoate--protein ligase OS=Staphylococcus aureus (strain NCTC 8325) GN=SAOUHSC_00963 P | 22.6588 | 24.9582 | | |
| sp\|Q9EZ12\|DAPA_STAA8 | 16.6 | 4 | 6 | 4-hydroxy-tetrahydrodipicolinate synthase OS=Staphylococcus aureus (strain NCTC 8325) GN=dapA PE=3 SV=1 | | | 25.6204 | 26.5945 |
| rev_tr\|Q2G1W8\|Q2G1W8_STAA8 | | 3 | 5 | | 27.8864 | 28.5495 | | 29.4068 |
| tr\|Q2FZ16\|Q2FZ16_STAA8 | 6.4 | 3 | 5 | Uncharacterized protein OS=Staphylococcus aureus (strain NCTC 8325) GN=SAOUHSC_01255 | 27.4741 | 27.2998 | 24.0746 | 23.7962 |
| tr\|Q2G009\|Q2G009_STAA8 | 83.3 | 3 | 5 | Uncharacterized protein OS=Staphylococcus aureus (strain NCTC 8325) GN=SAOUHSC_00819 | 27.4055 | 26.2495 | | |
| sp\|Q2FW38\|RL13_STAA8 | 33.1 | 5 | 5 | 50S ribosomal protein L13 OS=Staphylococcus aureus (strain NCTC 8325) GN=rplM PE=1 SV=1 | 26.2431 | 23.1632 | | |
| sp\|Q2FZW4\|DLTC_STAA8 | 26.9 | 3 | 5 | D-alanine--poly(phosphoribitol) ligase subunit 2 OS=Staphylococcus aureus (strain NCTC 8325 | 25.9294 | 27.5376 | | 24.6309 |
| tr\|Q2FZ70\|Q2FZ70_STAA8 | 22.2 | 4 | 5 | Orotate phosphoribosyltransferase OS=Staphylococcus aureus (strain NCTC 8325) GN=pyrE P | 25.4589 | 26.2972 | | |
| sp\|Q2FXU0\|APT_STAA8 | 24.4 | 3 | 5 | Adenine phosphoribosyltransferase OS=Staphylococcus aureus (strain NCTC 8325) GN=apt PE | 24.5393 | 24.4345 | | |
| tr\|Q2G2J5\|Q2G2J5_STAA8 | 8.9 | 3 | 5 | Uncharacterized protein OS=Staphylococcus aureus (strain NCTC 8325) GN=SAOUHSC_02569 | 24.1957 | 22.1791 | | 25.0328 |
| tr\|Q2FWX1\|Q2FWX1_STAA8 | 23.0 | 4 | 5 | Uncharacterized protein OS=Staphylococcus aureus (strain NCTC 8325) GN=SAOUHSC_02150 | 22.8966 | 23.6513 | 24.4364 | |
| tr\|Q2G0C9\|Q2G0C9_STAA8 | 14.6 | 3 | 5 | Uncharacterized protein OS=Staphylococcus aureus (strain NCTC 8325) GN=SAOUHSC_00676 PE=4 SV=1 | | | 25.9539 | 23.7375 |
| tr\|Q2FW95\|Q2FW95_STAA8 | 2.5 | 4 | 5 | Uncharacterized protein OS=Staphylococcus aureus (strain NCTC 8325) GN=SAOUHSC_02404 PE=4 SV=1 | | | 25.4018 | 25.5012 |
| tr\|Q2G205\|Q2G205_STAA8 | 6.2 | 3 | 5 | Gluconate kinase OS=Staphylococcus aureus (strain NCTC 8325) GN=SAOUHSC_02808 PE=3 SV=1 | | | 26.5877 | 26.601 |
| rev_sp\|Q2FYJ6\|EBH_STAA8 | | 4 | 4 | | 27.9919 | | 27.5237 | |
| tr\|Q2FVT5\|Q2FVT5_STAA8 | 11.9 | 3 | 4 | Urocanate hydratase OS=Staphylococcus aureus (strain NCTC 8325) GN=hutU PE=3 SV=1 | 27.9425 | 24.5128 | | |
| tr\|Q2FZY5\|Q2FZY5_STAA8 | 12.0 | 3 | 4 | Cysteine desulfurase OS=Staphylococcus aureus (strain NCTC 8325) GN=SAOUHSC_00849 PE= | 26.0333 | 23.9797 | | |
| sp\|Q2FYN2\|CSPA_STAA8 | 54.5 | 3 | 4 | Cold shock protein CspA OS=Staphylococcus aureus (strain NCTC 8325) GN=cspA PE=1 SV=1 | 25.788 | 23.8645 | | |
| tr\|Q2FVW7\|Q2FVW7_STAA8 | 14.6 | 4 | 4 | Uncharacterized protein OS=Staphylococcus aureus (strain NCTC 8325) GN=SAOUHSC_02556 | 25.7784 | 27.401 | | |
| sp\|Q2G0Q9\|HSLO_STAA8 | 13.7 | 3 | 4 | 33 kDa chaperonin OS=Staphylococcus aureus (strain NCTC 8325) GN=hslO PE=3 SV=1 | 25.3108 | 23.7671 | | |
| sp\|Q2FZ75\|PYRB_STAA8 | 9.9 | 3 | 4 | Aspartate carbamoyltransferase OS=Staphylococcus aureus (strain NCTC 8325) GN=pyrB PE=3 | 24.6561 | 24.1832 | 30.322 | |
| tr\|Q2FXV4\|Q2FXV4_STAA8 | 11.8 | 4 | 4 | Uncharacterized protein OS=Staphylococcus aureus (strain NCTC 8325) GN=SAOUHSC_01727 | 24.5642 | 22.7168 | | 23.5398 |
| sp\|Q2G1W4\|METK_STAA8 | 11.3 | 3 | 4 | S-adenosylmethionine synthase OS=Staphylococcus aureus (strain NCTC 8325) GN=metK PE= | 24.4357 | 23.0854 | | |
| tr\|Q2G2H4\|Q2G2H4_STAA8 | 10.1 | 3 | 4 | DNA polymerase III subunit beta OS=Staphylococcus aureus (strain NCTC 8325) GN=SAOUHSC | 24.4042 | 23.1334 | | |
| sp\|Q2FZ21\|RRF_STAA8 | 20.1 | 3 | 4 | Ribosome-recycling factor OS=Staphylococcus aureus (strain NCTC 8325) GN=frr PE=3 SV=1 | 24.2213 | 23.5327 | | |
| sp\|Q2FW18\|RL5_STAA8 | 19.0 | 4 | 4 | 50S ribosomal protein L5 OS=Staphylococcus aureus (strain NCTC 8325) GN=rplE PE=1 SV=1 | 23.8504 | 24.8521 | | |
| tr\|Q2FZI5\|Q2FZI5_STAA8 | 8.7 | 3 | 4 | Phosphoribosylamine--glycine ligase OS=Staphylococcus aureus (strain NCTC 8325) GN=purD | 23.3757 | 24.6656 | | |
| rev_contam_tr\|Q61869 | | 3 | 4 | | 22.8494 | 25.1961 | | 25.5317 |
| sp\|Q2FZV7\|Y878_STAA8 | 6.0 | 3 | 4 | NADH dehydrogenase-like protein SAOUHSC_00878 OS=Staphylococcus aureus (strain NCTC | 22.3956 | 23.7222 | 23.7787 | |
| tr\|Q2FV16\|Q2FV16_STAA8 | 7.4 | 3 | 4 | Probable malate:quinone oxidoreductase OS=Staphylococcus aureus (strain NCTC 8325) GN=mqo PE=3 SV=1 | | | 25.0549 | 24.1849 |
| sp\|Q2G055\|HPF_STAA8 | 15.8 | 3 | 4 | Ribosome hibernation promotion factor OS=Staphylococcus aureus (strain NCTC 8325) GN=hpf PE=1 SV= | | 27.6451 | 26.1942 | 24.7195 |
| sp\|Q9EZ11\|DAPB_STAA8 | 16.7 | 3 | 4 | 4-hydroxy-tetrahydrodipicolinate reductase OS=Staphylococcus aureus (strain NCTC 8325) GN=dapB PE=3 SV=3 | | | 25.8896 | 25.8469 |
| sp\|Q2FV23\|PAND_STAA8 | 32.3 | 3 | 3 | Aspartate 1-decarboxylase OS=Staphylococcus aureus (strain NCTC 8325) GN=panD PE=1 SV= | 24.4312 | 27.4654 | | |
| tr\|Q2FXM1\|Q2FXM1_STAA8 | 27.7 | 3 | 3 | Uncharacterized protein OS=Staphylococcus aureus (strain NCTC 8325) GN=SAOUHSC_01814 | 23.1037 | 24.1462 | | |
| tr\|Q2G1A9\|Q2G1A9_STAA8 | 7.7 | 3 | 3 | 6-phospho-beta-glucosidase, putative OS=Staphylococcus aureus (strain NCTC 8325) GN=SAO | 22.518 | 23.9216 | | |
| sp\|Q2FWK4\|ILVC_STAA8 | 12.6 | 3 | 3 | Ketol-acid reductoisomerase (NADP(+)) OS=Staphylococcus aureus (strain NCTC 8325) GN=ilvC PE=3 SV=1 | | | 25.8109 | |
